# Singlet fission mediates carotenoid-to-bacteriochlorophyll energy transfer in purple photosynthetic bacteria

**DOI:** 10.1038/s41557-026-02186-7

**Published:** 2026-06-23

**Authors:** Shuangqing Wang, George A. Sutherland, James P. Pidgeon, Mateja Šmitran, David J. K. Swainsbury, Elizabeth C. Martin, Cvetelin Vasilev, Andrew Hitchcock, Daniel J. Gillard, Ravi Kumar Venkatraman, Dimitri Chekulaev, Alexander I. Tartakovskii, C. Neil Hunter, Jenny Clark

**Affiliations:** 1 School of Mathematical and Physical Sciences, University of Sheffield, Sheffield, UK; 2 Department of Chemistry and Biochemistry, University of California, San Diego, La Jolla, CA USA; 3 School of Biosciences, University of Sheffield, Sheffield, UK; 4 School of Electrical and Electronic Engineering, University of Sheffield, Sheffield, UK; 5 School of Biological Sciences, University of East Anglia, Norwich, UK; 6 Department of Chemistry, SRM Institute of Science and Technology, Chengalpattu, India

**Keywords:** Energy transfer, Light harvesting, Excited states

## Abstract

Photosynthesis, the foundation of most life, begins when sunlight is captured by (bacterio)chlorophyll ((B)Chl) and carotenoid (Crt) pigments. These molecules are arranged so that captured energy migrates rapidly to reaction centres, where it is stored as a charge separation. The complementary absorption of Crt and (B)Chl pigments, and rapid energy transfer between them, underpins solar harvesting. Here we report a Crt-to-BChl energy transfer mechanism mediated by singlet fission, in which a high-energy singlet exciton (with spin quantum number *S* = 0) is converted into two low-energy triplet (*S* = 1) excitons. In purple photosynthetic bacteria, the Crt S_2_ singlet exciton splits into Crt and BChl triplet excitons on adjacent sites. Once formed, the triplets transfer cooperatively to BChl and onwards to reaction centres. Energy is transferred from a singlet Crt state, via the spin-protected long-lived triplet pair, to a singlet BChl state. Thus, this singlet fission-mediated mechanism augments solar energy harvesting for photosynthesis.

**Figure:**
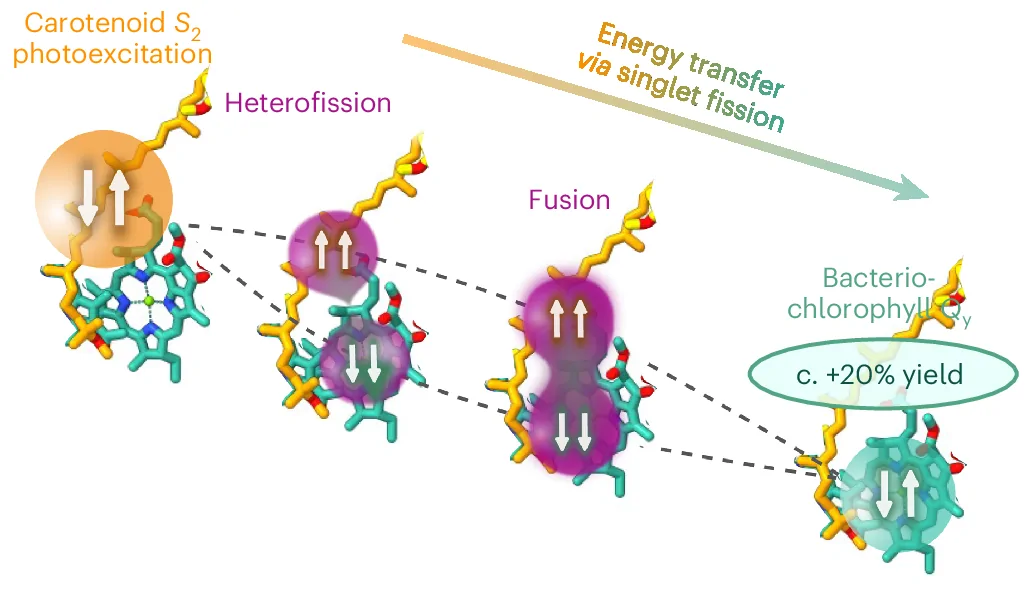

## Abstract (web summary)

The biological role of singlet fission—where two excitons are generated by one photon—is poorly understood. Now it has been shown that heterofission occurs in bacterial light-harvesting complexes, creating a triplet-pair reservoir that enhances carotenoid-to-bacteriochlorophyll energy transfer efficiency and temporarily staggers energy delivery to the reaction centre.

## Main

Singlet exciton fission (SF), the conversion of one spin-0 singlet exciton^1^ into a pair of spin-1 triplet excitons^2,3^, has been the focus of intense research in the past few years^4–7^. A remaining puzzle in the SF literature, however, is its role in biology^8–24^. Ultrafast sub-100-fs SF is known to occur in systems containing natural carotenoid (Crt) pigments^8–24^. These pigments form one of the most ubiquitous groups of organic molecules and are present in many organisms. Given their ubiquity, and known association with SF, it seems plausible that Crt-based SF may be occurring throughout the natural world. However, so far, only a few papers^11–24^ have discussed SF in natural systems or explored its biological function. Light-harvesting complexes (LHCs) from purple photosynthetic bacteria, for example, demonstrate surprisingly efficient SF when Crts absorb light^13–24^. Despite detailed study over the past 40 years, the mechanism and function of SF in these organisms, and the possible ramifications for phototrophs more widely, remain poorly understood. One puzzling aspect of reported SF in photosynthetic complexes, for example, is the ultrafast (sub-100 fs) formation of long-lived (>μs)^21^ triplet pairs, characteristics that are also ideal for SF-based technologies^25^ and difficult to achieve synthetically^26–32^.

**Fig. 1:**
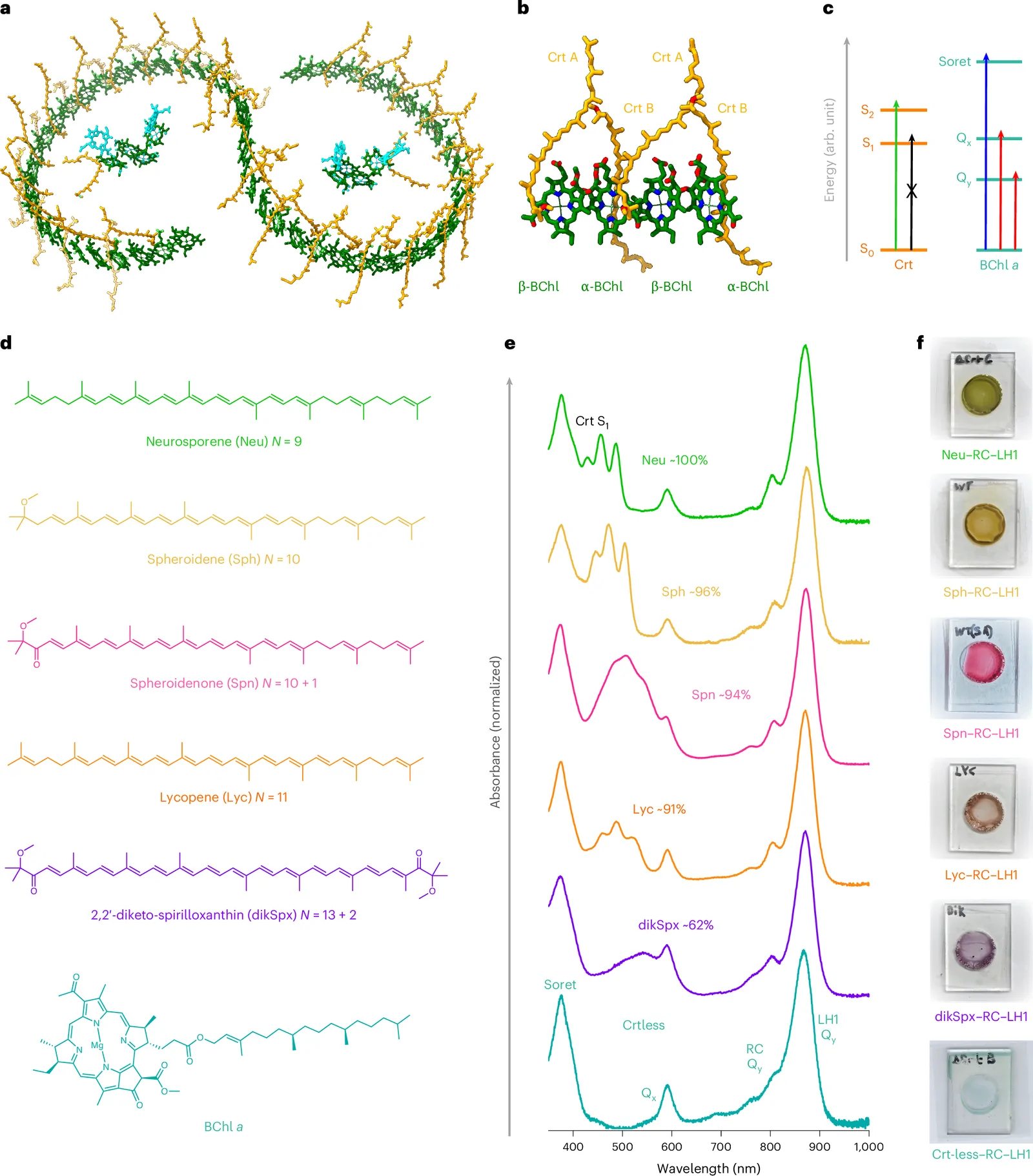
RC–LH1 complexes from *Rba. sphaeroides*. **a**, Atomic-resolution structure of the dimeric wild-type complex including antenna BChl *a* (green), Crts (orange) and RC pigments. Protein residues and the BChl *a* phytyl tail are omitted for clarity (PDBID: 7PQD, ref. ^35^). **b**, Arrangement of pigments in an adjacent pair of α−β subunits. Image rendering: ChimeraX^75^. **c**, Energy level schematic showing the lowest singlet states of Crt (orange) and BChl *a* (blue-green). Arrows (with cross) denote allowed (forbidden) optical transitions. **d**, Pigment chemical structures, names (acronyms) and conjugation lengths, *N*, (‘+*i*’ indicates conjugated C=O bonds). **e**, Absorption spectra of RC–LH1 complexes (optical path length 10 mm, OD 0.08 at 872 nm, *Q*_*y*_ peak). Percentages indicate the fraction of Crts in the RC–LH1 complex of the named Crt^37^. **f**, Images of RC–LH1 complexes in trehalose/sucrose glass. OD, optical density.

Here, we focus on isolated reaction centre (RC) light-harvesting 1 (RC–LH1) complexes from the purple photosynthetic bacterium *Rhodobacter* (*Rba*.) *sphaeroides*. Purple bacteria are often used as model systems to study fundamental photosynthetic processes, as their adaptable metabolism allows total removal or substitution of photosynthetic pigments and proteins without affecting viability. In addition, the structures of many RC–LH1 complexes have been determined at high resolutions^33–36^, and many mutagenic approaches have been described, including the genetic manipulation of Crts^37,38^. RC–LH1 complexes from *Rba. sphaeroides* are known to demonstrate SF^21^, and the wild-type pigment–protein chromophore structure, including the Crts, has been recently published^33^, reproduced in Fig. 1a,b. Using a combination of magneto-optical and transient spectroscopy on wild-type and engineered RC–LH1 complexes with varying Crt conjugation lengths, we show that SF proceeds via heterofission and contributes to Crt-to-bacteriochlorophyll (BChl) *a* energy transfer. Our findings reveal that, in RC–LH1 complexes containing Crts with conjugation lengths *N* ≈ 9−11, *S*_2_ photoexcitation rapidly generates pairs of triplet excitons on adjacent Crt and BChl *a* molecules. Delayed luminescence measurements (nanoseconds to milliseconds) indicate that these triplet pairs decay through geminate triplet–triplet annihilation (TTA), efficiently populating the lowest BChl *a* singlet state (*Q*_*y*_). Our findings establish a SF-mediated energy transfer pathway that enhances Crt-to-BChl *a* energy transfer yield and may help the organisms better ‘digest’ absorbed quanta by temporally spreading out their arrival time at the RC.

## Results

The RC–LH1 complexes shown in Fig. 1 were isolated, as described previously^38^, from wild-type cells (containing spheroidenone (Spn)) and from engineered strains^37^ (Fig. 1). The main pigments in these complexes are Crts and BChl *a*, whose low-energy singlet electronic states are shown in Fig. 1c, with chemical structures depicted in Fig. 1d. The absorption spectra of the RC–LH1 complexes (Fig. 1e) show Crt *S*_2_ absorption (450−550 nm) that red-shifts as the Crt conjugation length increases and three BChl *a* absorption bands. The Soret band absorbs around 390 nm, *Q*_*x*_ at ~600 nm and *Q*_*y*_ between 750 nm and 910 nm. The *Q*_*y*_ absorption feature is sensitive to structural changes^39^; thus, its similarity in the spectra in Fig. 1e, together with Crt resonance Raman measurements^21^ (Supplementary Fig. 1), suggests that the structure of the RC–LH1 complex remains the same, independent of the Crt species incorporated. We have chosen to include Spn–RC–LH1 in this series, despite the presence of an excited intramolecular charge transfer (ICT) state in Spn^40^, because Spn is the native Crt when *Rba. sphaeroides* is grown in semi-aerobic conditions^19^, making it biologically relevant.

To protect Crt-less–RC–LH1 and other complexes from photobleaching under laser illumination, samples were encapsulated in trehalose/sucrose glass^41–43^ (Supplementary Fig. 2).

### Singlet fission yield decreases as Crt conjugation length increases

**Fig. 2:**
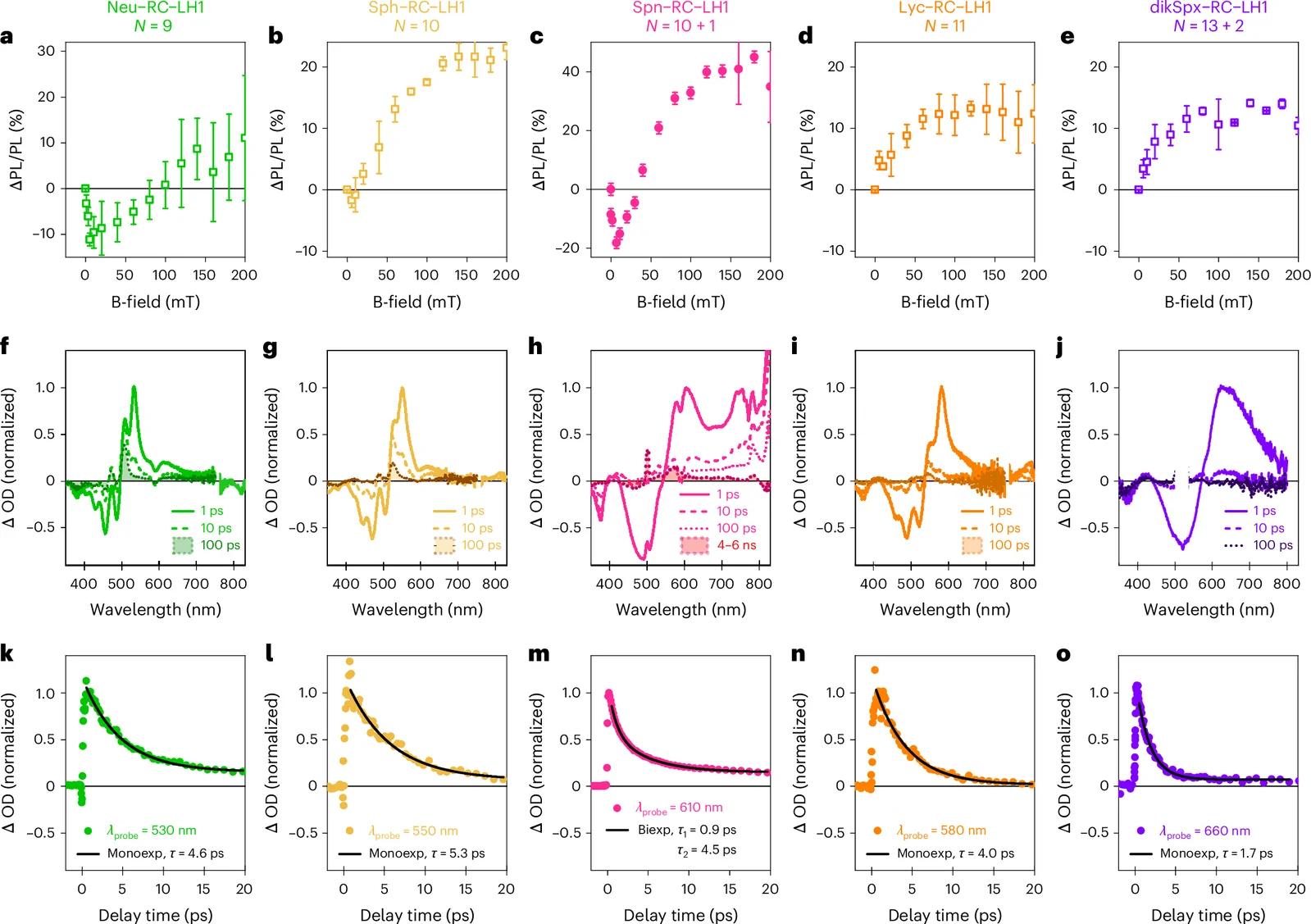
Spectroscopy of Crt–RC–LH1 complexes as a function of Crt conjugation length. **a**–**e**, MFE, measured as the relative change in PL intensity, normalized to zero-field for the Crt–RC–LH1 complexes marked in the titles. In **a**–**c**, the negative feature in the MFE demonstrates the presence of SF; in **d** and **e**, the signal is always positive and is instead dominated by RP behaviour (see text for details). Detection at 880 nm, 20−40 ns delay, excitation at *λ*_ex_ = 490 nm for Neu, *λ*_ex_ = 520 nm for others. Error bars: 2*σ* (standard deviation) from subsequent field up and down scans. **f**–**j**, TA spectra of Crt–RC–LH1 complexes indicated in the titles at Δ*t* = 1, 10, 100 ps and, for Spn–RC–LH1, Δ*t* averaged over 4−6 ns. The shaded spectra show triplets assigned to SF; the relative yield decreases with increasing conjugation length. *λ*_ex_ = 500 nm for Neu–RC–LH1 and *λ*_ex_ = 520 nm for others. **k**–**o**, TA dynamics at the peak of the *S*_1_ → *S*_*n*_ ESA band (markers) of the complexes indicated in the titles. Solid lines are mono-exponential fits, with lifetimes marked in the legend. Excitation conditions as in **f**–**j**. All complexes were measured following encapsulation in trehalose glass.

We begin by characterizing SF in these complexes using magnetic field-dependent photoluminescence (PL), a technique established for identifying SF and TTA in related systems^13,14^. Figure 2a–e shows the relative change in PL (ΔPL/PL = [PL(*B*) − PL(*B* = 0)]/PL(*B* = 0)) from the BChl *a* band at 880 nm under an applied field following Crt *S*_2_ excitation.

The magnetic field effect (MFE) profiles vary with Crt conjugation length. Complexes with shorter conjugation length Crts (neurosporene (Neu), spheroidene (Sph) and spheroidenone (Spn)) exhibit a characteristic low-field dip, followed by saturation above 125 mT. This profile is a hallmark of SF in systems with negligible intertriplet exchange interaction (*J* ≈ 0), implying minimal orbital overlap between the triplets in the nascent triplet pair^3,44^.

Physically, this *J* ≈ 0 regime reflects the energetic landscape of the nine triplet-pair substates. In the absence of exchange coupling, these states are nearly degenerate at zero field, perturbed only by the small (μeV) zero-field splitting arising from dipolar spin–spin interactions. Consequently, even modest magnetic fields are sufficient to lift this quasi-degeneracy and modulate the spin dynamics. By contrast, non-negligible exchange coupling (*J* ≠ 0) would split these states into pure-spin manifolds, shifting the field-induced state mixing to substantially higher magnetic fields (see the Supplementary Information and Supplementary Figs. 3–6 for details and simulations). Our *J* ≈ 0 MFE simulations show excellent agreement with the experimental MFEs for these short-chain complexes (Supplementary Fig. 6).

Conversely, complexes with longer conjugation length Crts (lycopene (Lyc) and 2,2′-diketo-spirilloxanthin (dikSpx) show a monotonic increase in ΔPL/PL with applied field, saturating above 50 mT. This behaviour arises from radical pair (RP) recombination within the RC^45–48^, suggesting negligible SF or TTA.

The observation of *J* ≈ 0 in SF-active complexes, and thus minimal wavefunction overlap between the triplets in the triplet pair, is particularly revealing. It challenges the conventional assumption that SF-generated triplets reside on a single chromophore, especially because no *π*-conjugation breaks are evident in the Crt backbone. Instead, the prompt BChl *a* triplet formation discussed below points towards a hetero-SF mechanism. In this model, a single Crt excited state (*S*_2_) produces triplet pairs partitioned between neighbouring Crt and BChl *a* molecules, naturally accounting for the spatial separation and the resulting vanishing exchange interaction.

To track the formation of these states directly, we turn to transient absorption (TA) spectroscopy with Crt S_2_ excitation (Fig. 2f–o). By 1 ps, all complexes exhibit Crt *S*_1_ → *S*_*n*_ excited state absorption (ESA) and ground state bleach (GSB) features, with *S*_1_ lifetimes (4–5.5 ps for *N* = 9−11) governed by competing non-radiative decay and *S*_1_ → *Q*_*y*_ energy transfer^49,50^. Notably, Spn–RC–LH1 also displays near-infrared (NIR) bands characteristic of an ICT state^19^ (we assume no ICT state in dikSpx–RC–LH1 as it is symmetric^40^).

Except for dikSpx–RC–LH1, all complexes also show long-lived Crt *T*_1_ → *T*_*n*_ features within 1 ps (Neu, 505 nm; Sph, 526 nm; Spn, 573 nm; Lyc, 547 nm), confirmed by comparison with sensitized triplet spectra^51–53^ (Supplementary Fig. 7). This sub-picosecond triplet formation aligns with our MFE results and independently confirms SF. In a departure from previous reports^18^, we find higher triplet yields for shorter conjugation length Crts (Supplementary Table 1).

The TA spectra also reveal BChl *a* features (bleaches at 378, 590 and 750–850 nm), indicative of Crt-to-BChl energy transfer within 1 ps. While these BChl *a* features are relatively weak, due to low excited state extinction coefficients of BChl *a* compared with Crts^54,55^ (Supplementary Fig. 8), the 750−830 nm region exhibits a distinct trend, transitioning from negative (triplet-associated bleach) in Neu–RC–LH1 to positive (singlet-associated absorption) in Lyc–RC–LH1. As discussed below, this inversion reflects the conjugation-length dependence of prompt BChl *a* triplet formation, providing evidence of heterofission.

### Hetero singlet fission generates triplets on neighbouring Crt and BChl *a* molecules

**Fig. 3:**
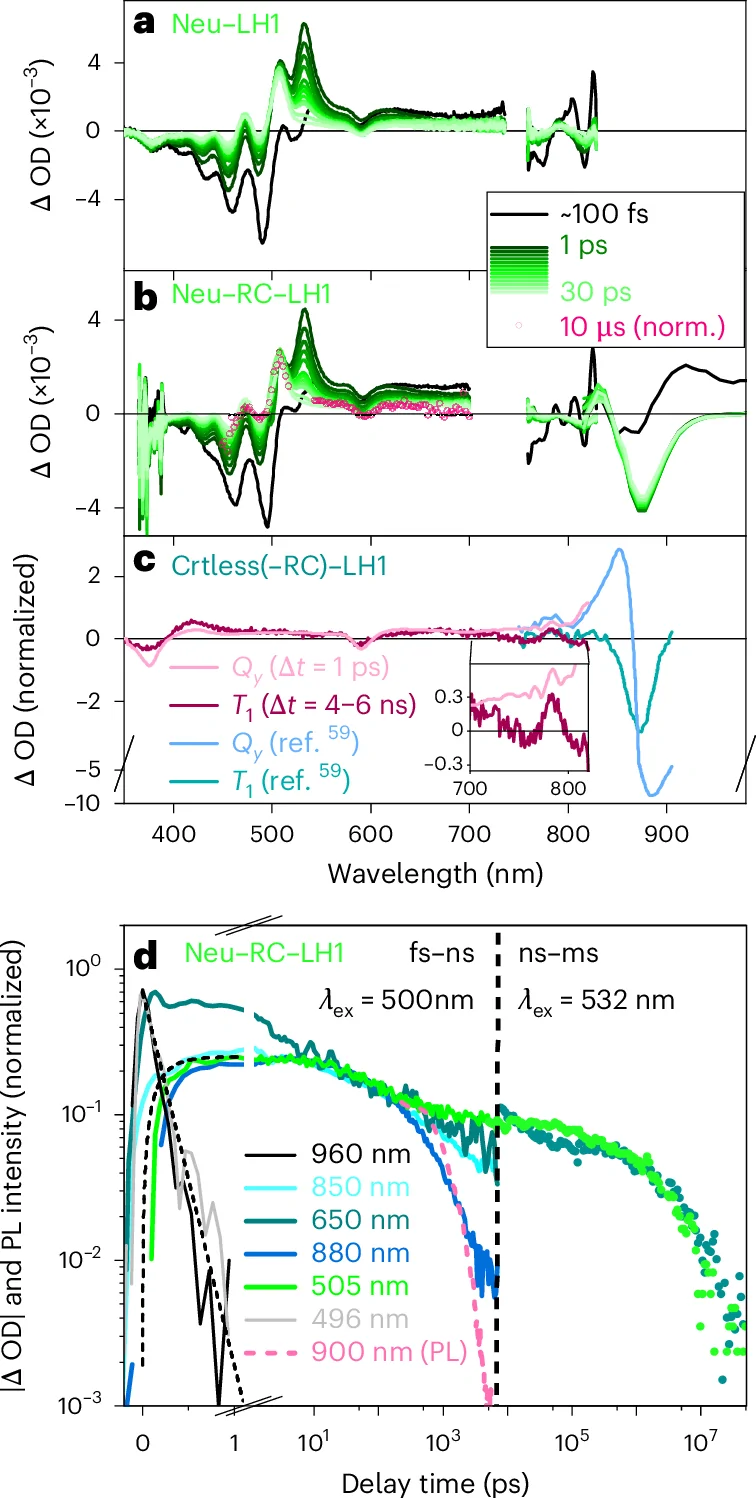
TA spectroscopy of Neu and Crt-less (RC–)LH1 complexes. **a**, TA spectra of Neu–LH1, *λ*_ex_ = 500 nm measured with two probe windows (ultraviolet–visible (UV–vis), 750−830 nm). Green spectra taken at Δ*t* = 1, 2, 3, 4, 5, 6, 8, 9, 10, 12, 20 and 30 ps delay time. **b**, TA spectra of Neu–RC–LH1 over three probe windows (UV–vis, 750−830 nm and 800−1,300 nm), Δ*t* = 10 μs spectrum normalized to the 30-ps spectrum (pink markers, *λ*_ex_ = 532 nm for the μs data); other details are as in **a**. **c**, BChl *a* TA spectra associated with *Q*_*y*_ and *T*_1_ population in a Crt-less–RC–LH1 measured in this work (*λ*_ex_ = 590 nm, pink) and replotted from ref. ^59^ (blue/green). Note the break in the *y*-axis scale. The inset shows a zoom-in of the fingerprint region: *T*_1_ has negative features (GSB), while *Q*_*y*_ is positive (ESA) (Supplementary Fig. 15). **d**, TA and PL transients of Neu–RC–LH1 complexes. Lines: fs–ns transients normalized at 700 ps (except 960 nm and 496 nm). Markers: ns–ms TA data normalized at 1 μs. Dashed black lines: monoexponential decay and rise, both with *τ* = 170 fs. Detection wavelengths marked in the legend. Crt-less–LH1 data in panel **c** from ref. ^59^.

To better assign the subtle BChl *a* features, we compare the TA spectra of a Crt-less RC–LH1 mutant (Fig. 3c) with those of the highest-yield complex, Neu(–RC)–LH1 (Fig. 3a,b). In the Neu-containing complexes, the earliest spectra exhibit stimulated emission (~496 nm) and NIR excited-state absorption characteristic of the *S*_2_ state. This population decays with a *τ* ≈ 170 fs lifetime (Fig. 3d), concurrently generating Neu *S*_1_ (520 nm), Neu *T*_1_ (505 nm) and BChl *a* (bleach at 378, 590 and 750–850 nm) populations^20^. Notably, the Neu–RC–LH1 spectral profile at 30 ps matches that recorded at 10 μs, indicating that BChl *a* triplets are formed on an ultrafast timescale (see Supplementary Fig. 9 for the full ns–ms spectra).

Distinguishing between BChl *a* singlet (*Q*_*y*_) and triplet (*T*_1_) populations from their TA spectra is notoriously difficult in the visible region because of their spectral similarity^56–58^ (Fig. 3c and Supplementary Figs. 10 and 11). This similarity is not an artefact of two-photon or sequential excitation effects, although these are also apparent (Supplementary Fig. 12). However, the NIR region (700–830 nm) provides a definitive fingerprint: as shown by the Crt-less(–RC)–LH1 TA spectra^59^ (Fig. 3c), the *Q*_*y*_ population yields a positive absorption, whereas *T*_1_ yields a negative bleach (Fig. 3, inset) (not a probe artefact; see Supplementary Figs. 13 and 14). Neu–RC–LH1 exhibits this characteristic *T*_1_ bleach alongside a dominant GSB at 870 nm, providing evidence that BChl *a* triplets are formed within 1 ps of Crt *S*_2_ excitation, a hallmark of hetero-SF (for a direct spectral comparison, see Supplementary Fig. 15).

The dynamics further support this assignment (Fig. 3d). Following the ~170 fs decay of the Neu *S*_2_ state, the BChl *a* *T*_1_ (650 nm, where Neu *T*_1_ should not absorb^23^) and Neu *T*_1_ (505 nm) features decay in tandem from 15 ns to 50 μs. We use single-wavelength analysis here, as global analysis revealed important correlations between species (see Supplementary Fig. 16 for details). Furthermore, the observed triplet half-lifetimes (*τ*_1/2_ ≈ 0.3–0.4 μs) are nearly an order of magnitude shorter than those of isolated triplets in ‘SF-inactive’ complexes (Lyc- and dikSpx–RC–LH1; Supplementary Fig. 9). This accelerated, correlated decay is characteristic of triplet-pair states undergoing TTA^10^.

We end our discussion of Fig. 3 by noting that the TA dynamics measured at 880 nm, near the peak of the GSB associated with the *Q*_*y*_ population, decay similarly to the *Q*_*y*_ PL (dashed line), with only a minor long-lived component, indicating that both transients are dominated by *Q*_*y*_ population decay. The presence of some *Q*_*y*_ population on picosecond timescales shows that *Q*_*y*_ states are also populated from Crt excitation via either direct singlet exciton energy transfer (EET), as described previously^22,50^, or rapid transfer from the triplet pair states to *Q*_*y*_. Because population of the *Q*_*y*_ state produces a strong GSB feature (more than 2.5× stronger than that of the BChl triplet population), any *Q*_*y*_ population is likely to dominate the BChl *a* GSB signatures in the NIR, making the weaker triplet population signatures difficult to observe^15^.

Taken together, these results indicate that SF in *Rba. sphaeroides* occurs via a heterofission mechanism^60^. In this model, Crt *S*_2_ excitation generates a delocalized triplet pair state, where the constituent triplets reside on neighbouring Crt and BChl *a* molecules, subsequently decaying in tandem via TTA.

### TTA populates BChl *aQ*_*y*_

**Fig. 4:**
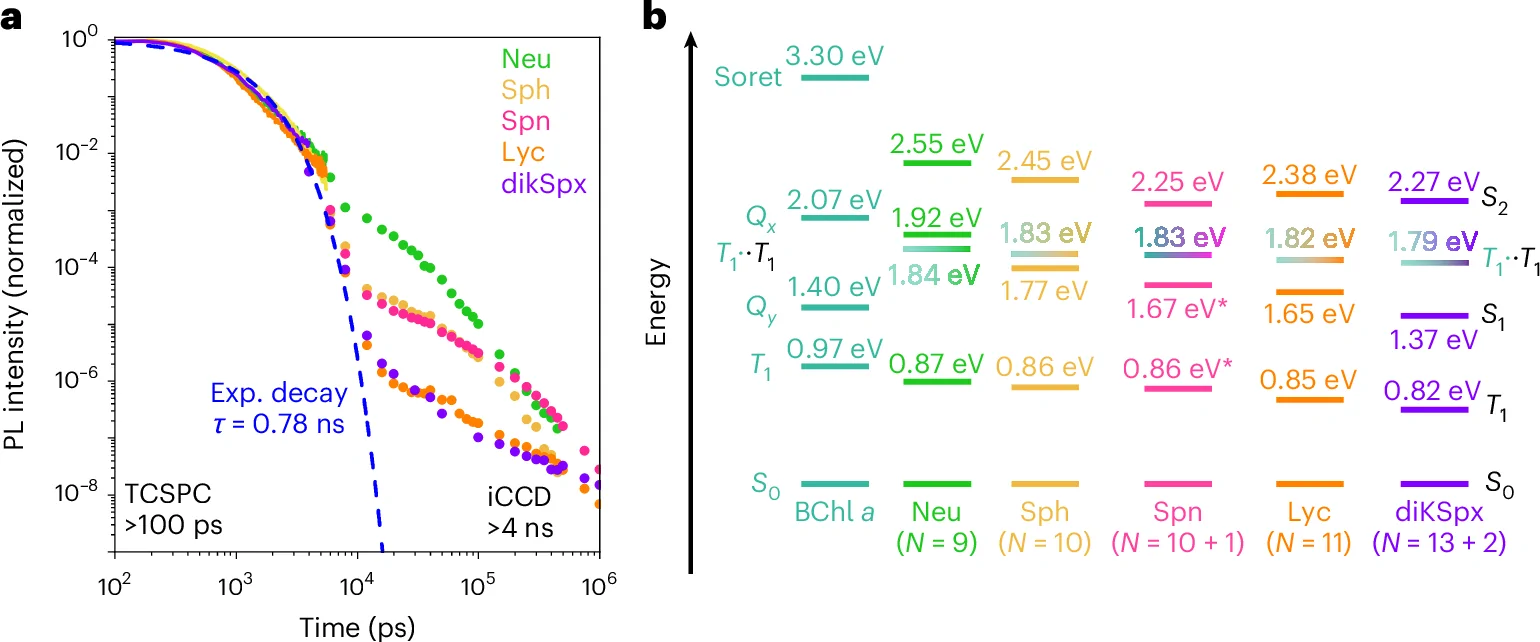
Fluorescence dynamics and energetics of RC–LH1 (*Rba. sphaeroides*) as a function of Crt conjugation length. **a**, Fluorescence measured in buffer to avoid scattering effects with Crt excitation (Neu, 500 nm; others, 520 nm), detection in the *Q*_*y*_ emission band (see Supplementary Fig. 17 for an example spectrum). Solid lines denote time-correlated single photon counting (TCSPC) measurements (normalized at 100 ps), markers denote gated intensified CCD measurements (normalized to TCSPC data) and the dashed line is a monoexponential function (Exp. decay) for comparison (*τ* = 0.78 ns). **b**, Approximate energies of BChl *a* and Crt excited states. Soret, *Q*-band and Crt *S*_2_ energies from absorption spectra, *T*_1_ and *S*_1_ energies, except Spn, from refs. ^76–79^. Spn *S*_1_ and *T*_1_ energies are assumed from the conjugation length dependence (Supplementary Table 3).

TTA is the microscopic reverse of SF, wherein a triplet pair fuses to regenerate a singlet excited state. Within the RC–LH1 architecture, TTA is expected to populate the *Q*_*y*_ state—the lowest-lying emissive singlet (Fig. 4b)—thereby generating delayed fluorescence on a 10–100 ns timescale. Notably, both bimolecular TTA and long-range triplet migration are excluded under our experimental conditions^61^ (Supplementary Fig. 16). Consequently, *Q*_*y*_ emission (850–900 nm) serves as a sensitive probe for the relaxation dynamics and energy transfer pathways following Crt excitation.

Figure 4a shows transient emission detected in the *Q*_*y*_ emission band (see example spectra in Supplementary Fig. 17). In all complexes, the prompt (<10 ns) BChl *a* *Q*_*y*_ emission decays identically, following a monoexponential function (dashed blue line). However, an extra delayed emission component is observed beyond 10 ns that is dependent on the conjugation length of the bound Crt. While delayed emission from RP recombination is a known feature of the RC^62^, it should remain largely invariant of the Crt species. Indeed, the Lyc- and dikSpx-containing samples exhibit a baseline delayed component that we attribute to RP recombination, consistent with our MFE assignments (Fig. 2d,e). By contrast, the SF-active complexes (Neu, Sph and Spn) exhibit an additional, high-amplitude delayed fluorescence component that scales directly with the triplet yield, confirming that the triplet pair state acts as a reservoir for the delayed *Q*_*y*_ population. We conclude that the shared triplet pair state, generated by Crt excitation, decays to *Q*_*y*_, providing an additional Crt-to-BChl *a* EET pathway.

Interestingly, the relaxed *S*_1_ energy is lower than the triplet pair state in all but the Neu-containing complex (Fig. 4b). Nevertheless, TTA to *Q*_*y*_ occurs in Sph- and Spn-containing RC–LH1 complexes, and appears not to be quenched by *S*_1_. One hypothesis to explain this finding is the fact that the *S*_1_ energy depends sensitively on the Crt conformation (for example, bond length alternation), which changes as the system relaxes^63,64^. We therefore speculate that TTA to *Q*_*y*_ is preferred over TTA to *S*_1_ because *S*_1_ is destabilized compared with *Q*_*y*_ at the *T*_1_ geometry^64^.

### Singlet fission as a mechanism for Crt-to-BChl *a* energy transfer

**Fig. 5:**
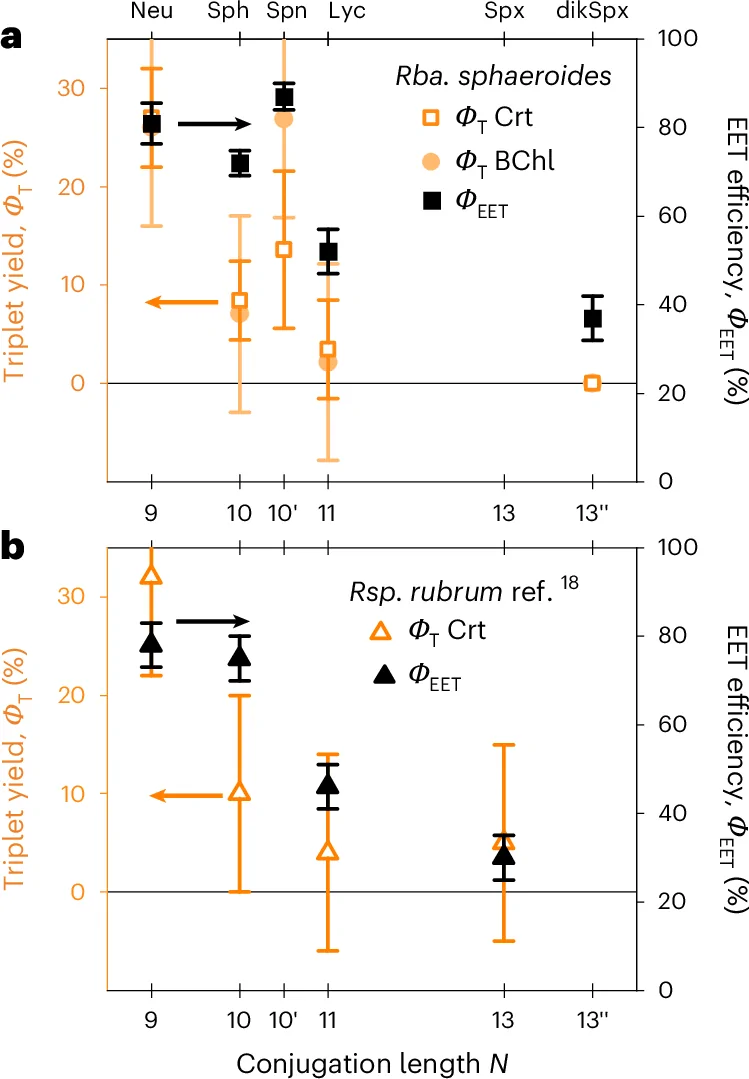
SF yield tracks crt-to-BChl *a* energy transfer efficiency. **a**,**b**, Triplet yield, *Φ*_T_, and Crt-to-BChl *a* EET efficiency, *Φ*_EET_, in Crt–RC–LH1 complexes as a function of Crt conjugation length (*N*) for *Rba. sphaeroides* (**a**) and *Rsp. rubrum* (**b**). See details in text and Supplementary Tables 1 and 2. The general trend, with the exception of Spn–RC–LH1 (which is the only Crt–RC–LH1 studied here to demonstrate an ICT state^19^), is a reduction in both triplet yield and EET efficiency with increasing Crt conjugation length. Note that complexes with high triplet yield show *Φ*_T_ + *Φ*_EET_ > 100%, indicating that SF contributes to EET. Errors are estimated from fitting errors, 2*σ* spread or variability in the literature. Data in **b** replotted from ref. ^18^.

We showed above that SF occurs via heterofission in Crt–RC–LH1 with *N* = 9−11, and the reservoir of SF-generated triplets populates BChl *a* *Q*_*y*_ via TTA. Thus, SF mediates energy transfer between Crts and BChls *a*. To determine the importance of this energy transfer pathway, in Fig. 5 we compare the triplet yield (*Φ*_T_) after Crt excitation with the Crt-to-BChl *a* EET efficiency (*Φ*_EET_) as a function of Crt conjugation length. Figure 5a shows results for *Rba. sphaeroides*, whereas Fig. 5b shows those for *Rhodospirillum* (*Rsp*.) *rubrum*^18^.

The triplet yields were obtained from TA spectra in Fig. 2 (*Rba. sphaeroides*) and ref. ^18^ (*Rsp. rubrum*) using published Crt and BChl *a* extinction coefficients^54,55,65^ (see Supplementary Table 2 for details). We note that the absolute Crt triplet yields reported here are generally lower than previous reports^13–15,21,66^, but the trend is robust. As the conjugation length increases, the yield of both Crt and BChl *a* triplets decreases. The close correlation between Crt and BChl *a* triplet yields further confirms heterofission as the SF mechanism. The trend of decreasing triplet yield with increasing conjugation length is consistent across both *Rba. sphaeroides* (squares) and *Rsp. rubrum* (triangles), despite the latter containing only half the Crt density^67^. This reinforces the conclusion that SF does not involve Crt–Crt interactions. Instead, these findings suggest that heterofission is a general phenomenon in purple bacteria, occurring specifically between the Crt (Crt A in Fig. 1b) and its neighbouring BChl *a* monomer—the only available partner in the *Rsp. rubrum* architecture.

The Crt-to-BChl *a* EET efficiency, *Φ*_EET_, shown in Fig. 5a, was quantified by comparing 1 − transmittance and fluorescence excitation spectra, as described previously^18,22,37,38,68^ (see Supplementary Fig. 18 for details). Reported values for *Rsp. rubrum* were taken directly from ref. ^18^.

The figure indicates that there is a correlation between *Φ*_T_ and *Φ*_EET_ and that, apart from Spn–RC–LH1, which has substantial ICT character, both the SF yield and EET efficiency drop with increasing conjugation length. Crucially, for the shorter Crts, the sum of *Φ*_T_ and *Φ*_EET_ exceeds 100%. This observation confirms our hypothesis that SF contributes to Crt-to-BChl *a* EET.

To determine the relative importance of this pathway, we estimated relative yields of all Crt-to-BChl *a* energy transfer pathways (Supplementary Table 1). Our analysis reveals that, for Neu–RC–LH1, approximately 18(8)% of the total Crt-to-BChl *a* energy transfer is mediated by the SF pathway.

## Discussion

We propose that SF-mediated energy transfer offers a mechanism to circumvent rapid, non-radiative dissipation of Crt singlet states. By transiently ‘storing’ the electronic energy in triplet-pair states, the system effectively bypasses the femtosecond thermalization pathways that typically limit light-harvesting efficiency. While individual triplets are too low in energy to contribute usefully to energy transfer, the combined energy of the Crt–BChl *a* triplet pair can be harvested cooperatively via TTA to *Q*_*y*_. This pathway leverages the spin-protected nature of the triplet state to enhance Crt-to-BChl energy transfer yield, allowing the organism to more effectively use the green/blue solar spectrum.

Beyond increasing absolute quantum yields, this heterofission pathway introduces a temporal staggering of energy delivery to the RC. As indicated by the delayed emission in Fig. 4, energy reaches the RC through two distinct temporal channels: a prompt singlet channel and a delayed triplet-pair reservoir. This ‘energy buffering’ may protect the RC during transient fluctuations in light intensity, effectively distributing the arrival of excitons to match the RC’s processing capacity.

The utility of such a scheme hinges on a difficult physical compromise: SF must be ultrafast (sub-picosecond) to compete with internal conversion, yet the resulting triplet pairs must not undergo rapid geminate recombination to the ground state. This combination is unusual: ultrafast SF is almost always associated with rapid triplet-pair decay, in the absence of triplet migration^26,28,30,69,70^, due to the strong electronic couplings involved^2,3,71^.

Our results indicate that heterofission—forming triplet pairs directly from *S*_2_ within a few hundred femtoseconds (Fig. 3d)—provides a potential solution to this problem. This direct *S*_2_-to-triplet-pair formation also explains the conjugation-length-dependent triplet yield (Fig. 5), as SF must compete with *S*_2_ → *S*_1_ internal conversion^49^ and energy transfer to *Q*_*x*_, the rates of which are strongly dependent on conjugation length^49,50^. While the initial fast formation of triplet pairs results from strong exchange coupling, the measured triplet pairs are weakly coupled (Fig. 2) and long-lived (Fig. 3). We propose that dynamic localization, driven by heterofission and strong vibronic coupling, rapidly decouples the triplets by minimizing orbital overlap and exchange coupling^72^. This process, akin to relaxation along the combined Crt–BChl potential energy surface, generates long-lived, weakly coupled triplet pairs necessary for efficient energy transfer—offering a biological alternative to triplet migration strategies used in synthetic systems^28,30^.

## Conclusion

In this work, we have demonstrated that the sophisticated structure of purple bacteria LHCs enables both ultrafast SF and the generation of long-lived triplet excitons through a process known as heterofission, involving neighbouring Crt and BChl pigments. These long-lived triplets act as an energy reservoir, temporarily storing energy to avoid rapid thermal dissipation, enhancing Crt-to-BChl energy transfer by up to 20%.

While SF has been predominantly observed in photosynthetic purple bacteria, which often inhabit low-light anaerobic environments where efficient light harvesting is crucial, the widespread presence of Crts and (B)Chls across diverse photosynthetic organisms suggests that similar SF mechanisms may be occurring in other natural systems. Furthermore, the ability of photosynthetic LHCs to dynamically decouple triplet pairs through heterofission, or potentially even to tune between SF and charge transfer by altering pigment–protein structure, offers new strategies for understanding SF and for designing^73^ new materials for SF-inspired applications^7,74^.

## Methods

### Preparation of RC–LH1 complexes containing a series of Crts

*Rba. sphaeroides* was grown and RC–LH1 complexes were purified according to our method previously described at length^38^. In brief, for wild-type and mutant stains, a single colony from an M22 agar plate was used to inoculate a 10-ml universal medical specimen tube containing M22 liquid medium. This was incubated in the dark at 34 °C with agitation (150 rpm) until visibly turbid. This culture was then scaled up to 80 ml, also in liquid M22 medium, in 125-ml Erlenmeyer flasks. Cells were then either cultured by semi-aerobic heterotrophic growth or anaerobic phototrophic growth, as described below.

For semi-aerobic heterotrophic growth, the 80-ml culture was added to 1.6 litres of M22 liquid medium in a 2-litre Erlenmeyer flask and incubated at 34 °C with agitation (150 rpm). For anaerobic phototrophic growth, 1 litre of M22 medium in a 1.2-litre medical flat was sparged with filtered ultrapure N_2_ under aseptic conditions. The 80-ml culture was added, and the flask was filled with additional sparged M22 medium. Flasks were then sealed gently with a rubber stopper and parafilm, agitated with a magnetic stirrer and illuminated with white light (50−500 μmol photons m^−2^ s^−1^) at room temperature.

Cells were collected by centrifugation (4,400*g*, 30 min, 4 °C), resuspended in resuspension buffer (20 mM HEPES, pH 8.0, and 5 mM EDTA) and lysed by two passes through a French pressure cell (18,000 psi, precooled to 4 °C). This solution was centrifuged (27,000*g*, 15 min, 4 °C), and the supernatant was collected. Membranes were obtained by loading the supernatant onto 15/40% (w/v) sucrose step gradients, prepared in the appropriate tubes for a Beckman Ti45 fixed angle rotor, and centrifuged at 85,000*g*, 4 °C for 10−16 h. Chromatophore membranes were collected from the interface between the two sucrose solutions, diluted sixfold in resuspension buffer and centrifuged at 185,000*g* for 2 h. All supernatant was removed and membrane pellets were resuspended and homogenized in 5 ml resuspension buffer. *N*-dodecyl-β-D-maltoside (β-DDM) was added to a final concentration of 2% (w/v), and membranes were solubilized at 4 °C for 1 h.

Solutions of resuspension buffer containing 20%, 21.25%, 22.5%, 23.75%, 25% and 50% (w/w) sucrose were prepared in tubes appropriate for a Beckman SW41 Ti rotor. Solubilized membranes were loaded on top of the gradient and centrifuged at 125,000*g* at 4 °C for 40 h. The appropriate band for RC–LH1 dimers (or monomers where appropriate) was collected with a fixed needle.

Protein samples were further purified by anion exchange chromatography using a 5-ml Q-sepharose HP column (Cytiva) and a linear gradient of buffer A (50 mM HEPES, pH 8.0, 0 mM NaCl and 0.03% (w/v) β-DDM) to buffer B (50 mM HEPES, pH 8.0, 1 M NaCl and 0.03% (w/v) β-DDM) over 60 column volumes. Appropriate fractions were pooled, concentrated by centrifugal dialysis and loaded onto a Superdex 200 Increase 10/300 GL size exclusion chromatography column (Cytiva), pre-equilibrated in 50 mM HEPES, pH 8.0, 100 mM NaCl and 0.03% (w/v) β-DDM. Apart from the Crt-less RC–LH1 sample, fractions with an A875/A280 ratio >1.4 were pooled for analysis.

For deposition in trehalose/sucrose films, the RC–LH1 samples were concentrated to give A875 values of 10−20 (apart from Crt-less RC–LH1, which was approximately 0.5). Then, 40 μl of sample was mixed with 40 μl of trehalose/sucrose mix (both 0.5 M) and 5 mM sodium ascorbate. Two imaging spacers (Secure Seal diameter 9 mm, thickness 0.12 mm; Grace Bio-Labs) were then stacked on a quartz-coated glass substrate (Ossila), and the RC–LH1 trehalose solution was deposited in the centre of the spacer. The substrate was placed in a vacuum chamber (−70 kPa) with a large excess of calcium sulfate desiccant (Drierite) and dried for a minimum of 3 days. Following drying, a No. 1 coverslip was applied to the imaging spacer to protect the glass from atmospheric rehydration.

### Static spectroscopy

Static absorption spectra were carried out using an Agilent Cary 60 spectrometer. Static fluorescence excitation spectra were acquired with a Jobin–Yvon Fluorolog–3 spectrofluorometer equipped with a Hamamatsu FL-1030 cooled photomultiplier. The spectra were recorded at 898 nm with excitation and emission slits set to 3 and 10 nm, respectively. To minimize reabsorption, the optical density at 872 nm for each sample in buffer solution was adjusted to <0.1. The fluorescence excitation spectra were collected with a short exposure time and multiple accumulations (~7 h in total).

The resonance Raman spectra were measured with film samples at room temperature using a free-space optical set-up. In brief, the spectra were recorded via an SP750 spectrograph (Princeton Instruments) equipped with a holographic 1,800 grooves mm^−1^ grating and a back-illuminated liquid-nitrogen-cooled charge-coupled device (CCD; Princeton Instruments, PyLon). Excitation at 532 nm was provided by a single-mode diode-pumped solid-state laser (Cobolt, 04-01) with power of 1 mW focused in a spot of 2 μm diameter on the sample and spectral linewidth of <1 MHz. The spectral resolution of the system was ~0.4 cm^−1^.

### TA spectroscopy

The sub-picosecond TA spectra were recorded utilizing a Ti:Sapphire laser system (Spitfire ACE PA-40, Spectra-Physics) delivering pulses with pulse lengths of 40 fs (full width at half maximum) at 800 nm and a repetition rate of 10 kHz. Pump pulses at 500 nm (and 520 nm) were generated in an optical parametric amplifier (TOPAS Prime, Light Conversion). Probe pulses were obtained by supercontinuum generation (SCG) in CaF_2_ using the 800-nm Ti:Sapphire fundamental and the fundamental alone. The pump–probe time delay was set using a computer-controlled multipass motorized linear delay stage. The pump polarization was set at the magic angle (54.7°) relative to the probe polarization. After the sample film, the probe pulses were detected by using a commercial instrument (Helios, Ultrafast Systems) equipped with a complementary metal–oxide–semiconductor detector for the 340−830 nm spectral region.

The nanosecond broadband absorption set-up was based on a 90-fs, 1-kHz Ti:Sapphire amplifier (Solstice, Spectra-Physics). Broadband supercontinuum probe pulses between 450 nm and 700 nm were generated in a sapphire plate. Pump pulses were provided by an Nd:YVO4 Q-switched laser (Innolas Picolo-AOT) tuned for second-harmonic (532 nm) output. The pump–probe delay was controlled electronically using a digital delay generator (DG645, Stanford). The polarization of the pump was set to the magic angle (54.7°) with respect to the polarization of the probe. Signal and reference probe pulses created by a beamsplitter were detected spectrally resolved pulse by pulse by two CCD cameras (S7030, Hamamatsu) driven and read out at the full laser repetition rate by a custom-built board from Entwicklungsbüro Stresing.

### Time-resolved PL spectroscopy

The BChl *a* Q_y_ fluorescence lifetime of the RC–LH1 complex was measured on a home-built time-resolved fluorescence microscope. The microscope was equipped with a 485-nm picosecond diode laser (PicoQuant, PDL 828) as an excitation source. The excitation light was focused by a 100× objective (PlaneFluorite, numerical aperture 1.4, oil immersion, Olympus). The emitted light was filtered using a 495-nm dichroic beamsplitter (Semrock) and 900/32 nm bandpass filter (Semrock) to remove the background excitation light. The microscope was fitted with a spectrometer (150 lines mm^−1^ grating, Acton SP2558, Princeton Instruments) and an electron-multiplying CCD camera (ProEM 512, Princeton Instruments) for emission spectrum acquisition. A hybrid detector (HPM-100-50, Becker & Hickl) was used for single-photon counting. The modulation of the excitation laser was synchronized with a time-correlated single-photon counting (TCSPC) module (SPC-150, Becker & Hickl) for the lifetime decay measurement. The repetition rate of the laser was set at 1 MHz. The excitation laser power was adjusted to produce a fluence of approximately 2 × 10^14^ photons per pulse per square centimetre. The instrument response function of the set-up was approximately 130 ps.

The nanosecond–millisecond time-resolved PL spectra were recorded via a time-gated intensified CCD (iCCD; iStar DH334T-18U-73, Andor) coupled to a Shamrock 303i spectrograph. Excitation pulses at 490 and 520 nm were generated from a home-built non-collinear optical parametric amplifier (NOPA) pumped by a 1-kHz regeneratively amplified 800-nm Ti:Sapphire laser (Solstice, Spectra-Physics).

### Magnetic field-applied PL spectroscopy

The MFEs on the PL of the RC–LH1 complex were measured by recording the PL intensity changes at a series of magnetic field strengths (0−300 mT). The film samples were placed between the poles of a magnet (Newport Instrument, serial no. 8248/II). Excitation pulses at 490 and 520 nm were created from a home-built NOPA. The PL was detected at a wavelength of ~880 nm with a delay time of 20–40 ns using a spectrograph (Shamrock 303i, Andor) and a time-gated iCCD (iStar DH334T-18U-73, Andor). A 830-nm longpass filter (FEL830, Thorlabs) was placed before the detector to cut out scattered pump light. The MFEs on the RC–LH1 complex were evaluated as $\frac{\Delta {\mathrm{PL}}}{\mathrm{PL}}=\frac{{\mathrm{PL}}_{B}-{\mathrm{PL}}_{0}}{{\mathrm{PL}}_{0}}$.

## Online content

Any methods, additional references, Nature Portfolio reporting summaries, source data, extended data, supplementary information, acknowledgements, peer review information; details of author contributions and competing interests; and statements of data and code availability are available at https://doi.org/10.1038/s41557-026-02186-7.

## Supplementary information


Supplementary InformationSupplementary Figs. 1–18, Tables 1–3 and Note ‘Magnetic Field Effects’.


## Data Availability

The datasets underlying figures in this Article and the Supplementary Information are available via the University of Sheffield’s ORDA repository (hosted by Figshare) at https://doi.org/10.15131/shef.data.32013198 (ref. ^80^). Biological materials are available on request.
