# Supplementary material for: Singlet fission mediates carotenoid-to-bacteriochlorophyll energy transfer in purple photosynthetic bacteria
Source: Nat Chem. 2026 Jun 23;18(9):1611–9. doi: 10.1038/s41557-026-02186-7 (PMC13524793; doi:10.1038/s41557-026-02186-7)
Supplement: Supplementary file 1 [file 41557_2026_2186_MOESM1_ESM.pdf]

nature chemistry

Supplementary information https://doi.org/10.1038/s41557-026-02186-7

Singlet fission mediates carotenoid-to-bacteriochlorophyll energy transfer in purple photosynthetic bacteria

In the format provided by the authors and unedited

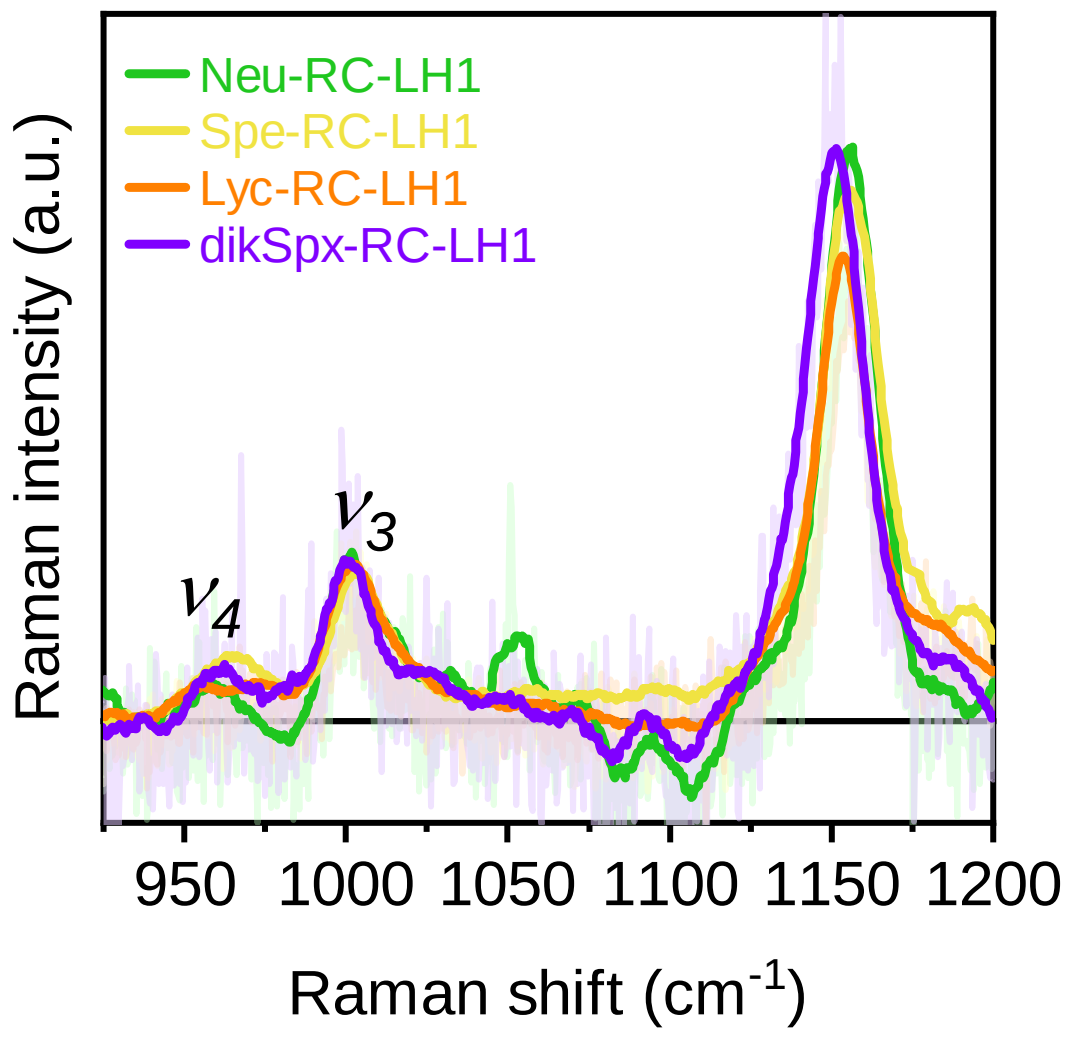

Fig. S1: Resonance Raman (RR) spectra in the fingerprint region ($\sim$ 960 cm^{-1}) of Crt–RC–LH1 complexes in trehalose films containing neurosporene (Neu), spheroidene (Spe), lycopene (Lyc), and 2,2'-diketo-spirilloxanthin (dikSpx) upon excitation at $\lambda_{\text{ex}} = 532$ nm. The data have been background-subtracted (faint lines), normalised to the ν_3 peak at $\sim$ 1000 cm^{-1} and smoothed (thick lines) using an Adjacent Averaging filter (30 points). Note that owing to the scatter from the films, the background subtraction is not perfect, particularly for Neu–RC–LH1. All four Crts in the RC–LH1 complex measured here show a similar fingerprint region, with the same relative ν_4 amplitude compared with ν_3, within experimental error, suggesting that the Crts are maintained in a similar, twisted, configuration within the RC–LH1 complexes.

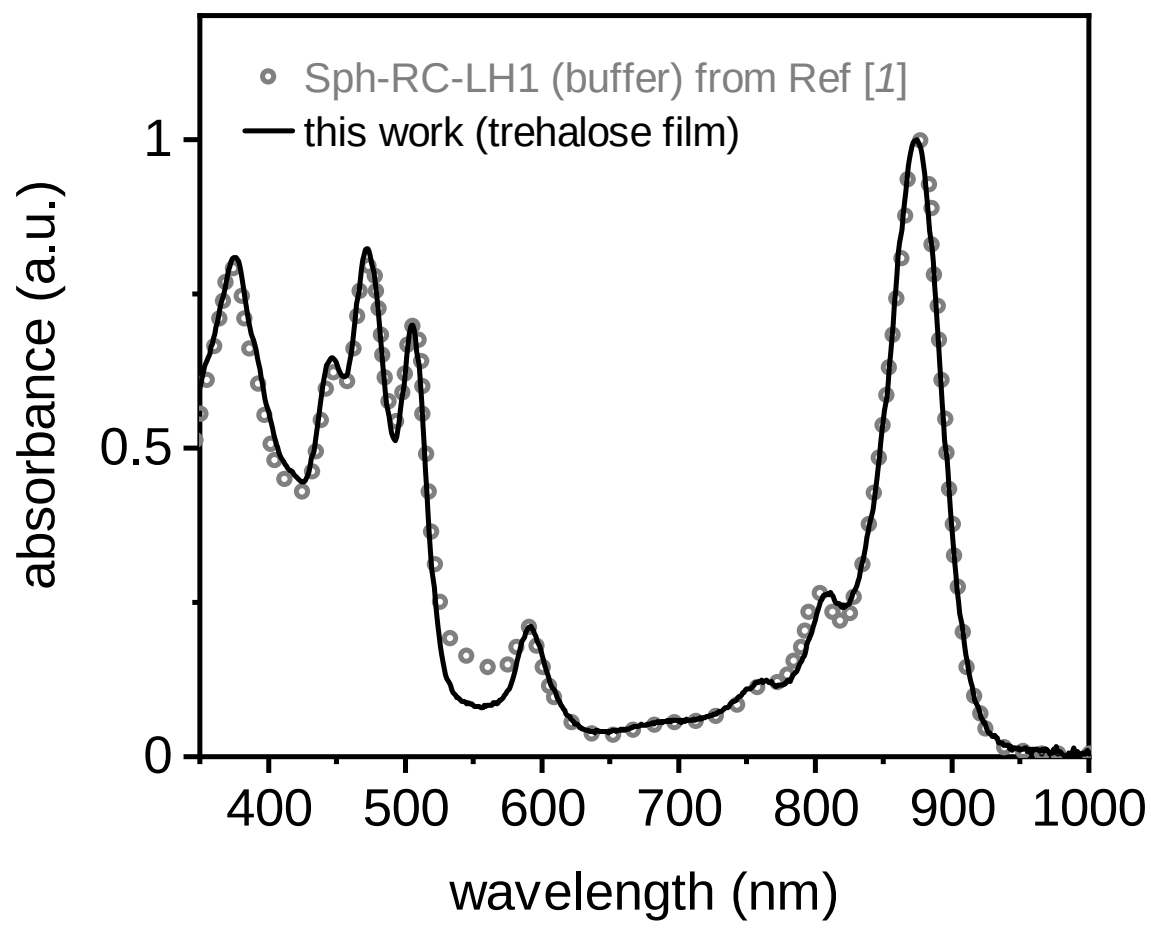

Fig. S2: Static absorption spectra of the RC–LH1 complex containing spheroidene in a trehalose film (line) and in buffer solution (circles) [1] (used with permission). The static spectra of the Sph–RC–LH1 complex either encapsulated in trehalose films or dissolved in buffer solutions are similar, indicating that the solid trehalose film does not affect the photophysical properties of RC–LH1 complexes. Note that light scatter is often observed in the 350 − 600 nm range as precisely controlling the glass thickness, and hence path length, is not possible using the above method. Here the Q_y absorption band of RC–LH1 complexes from *Rba. sphaeroides* is at 872 nm, instead of usually reported 875 nm. To confirm the reproducibility of this spectral feature we prepared these mutant complexes by two different methods: photosynthetically and semi-aerobically, and both batches show the same 872 nm Q_y band. We note that despite the shift, the ratio between the 872 nm and 275 nm bands is the same as in previous work: $\sim$ 1.6.

Magnetic Field Effects

The Three Triplet States

What we typically call a triplet state in the main text, T_1, is in fact a cluster of three individual states. These can be written in the 'zero-field basis', defined with respect to the molecular frame (a coordinate system fixed to the molecule), as $|T_x\rangle$, $|T_y\rangle$, and $|T_z\rangle$. In organic molecules, x is typically along the long axis of the molecule, y along the short axis, and z is perpendicular to the conjugated plane. See the excellent description in Section 3 of Ref. [2] for more information.

At zero magnetic field, these states have slightly different energies due to the so-called zero-field (ZF) interaction, mainly determined by spin-spin coupling. In most triplet states of organic compounds, the spin-orbit and crystal-field contributions to the ZF interaction can be neglected, and only the spin-spin contribution needs to be considered. The Hamiltonian can then be expressed in analogy to the classical dipole-dipole interaction and characterised by the ZF interaction tensor $\mathbf{D}$. As $\mathbf{D}$ is a traceless tensor, it can be represented by two parameters: D and E, where $|D| > 3|E|$ by convention. D is related to the average distance between the triplet's spins, while E reflects the rhombicity of the ZF interaction tensor. Their relationship to the energies of the triplet states is given by:

$$\begin{aligned} E_{T_x} &= -D_x = \frac{1}{3}D - E \\ E_{T_y} &= -D_y = \frac{1}{3}D + E \\ E_{T_z} &= -D_z = -\frac{2}{3}D \end{aligned} \tag{1}$$

The Nine Triplet-Pair States

In singlet fission (SF), the initial product can be approximated as a *pair* of triplets: $S_0S_1 \rightleftharpoons (T_1T_1)^l$ where S_1 represents a generic HOMO→LUMO type singlet excited state, and l labels the triplet-pair states.

There are *nine* distinct triplet-pair states, labelled by l. These triplet-pair states can be obtained using basis states written as combinations of the original triplet states (e.g., $|T_xT_x\rangle$, $|T_xT_y\rangle$, $|T_xT_z\rangle$, $|T_yT_y\rangle$, etc.) and diagonalising the spin Hamiltonian below. This provides eigenstates and energies of the triplet-pair states as a function of magnetic field. The spin Hamiltonian is given by:

$$\begin{aligned} \hat{H} = \sum_{i=a,b} &\left[g\mu_B \mathbf{B}\cdot\hat{\mathbf{S}}_i + D\left(\hat{S}_{i,z}^2 - \frac{1}{3}\hat{S}_i^2\right) + E\left(\hat{S}_{i,x}^2 - \hat{S}_{i,y}^2\right)\right] \\ &+ J\hat{\mathbf{S}}_a\cdot\hat{\mathbf{S}}_b + X\hat{\mathbf{S}}_a\cdot\hat{\mathbf{S}}_b, \end{aligned} \tag{2}$$

where g is the Landé g-factor, μ_B is the Bohr magneton, $\mathbf{B}$ indicates the applied magnetic field, and D and E are the zero-field splitting (ZFS) parameters for the individual triplets, as described above. $\mathbf{S}_i$ is the spin operator of the triplets (on

chromophores a and b), J is the exchange coupling between the two triplets and depends sensitively on the orbital overlap between them (equivalently described by considering the coupling of the triplet-pair state to an intermolecular charge-transfer (CT) state). X represents the inter-triplet spin-spin dipolar interaction. In this case, we ignore hyperfine interaction and spin-orbit coupling terms for simplicity [3].

Fig. S3 shows the nine triplet-pair eigenstates as a function of magnetic field magnitude, $|\mathbf{B}|$, using the following parameters: $D = 4$ μeV, $E = 0.5$ μeV from Ref. [4], $X = D/10000$, $g = 2$, and values of J as marked in the figures. The figure illustrates how the energies of the nine triplet-pair eigenstates vary with the applied magnetic field for different values of the exchange coupling J.

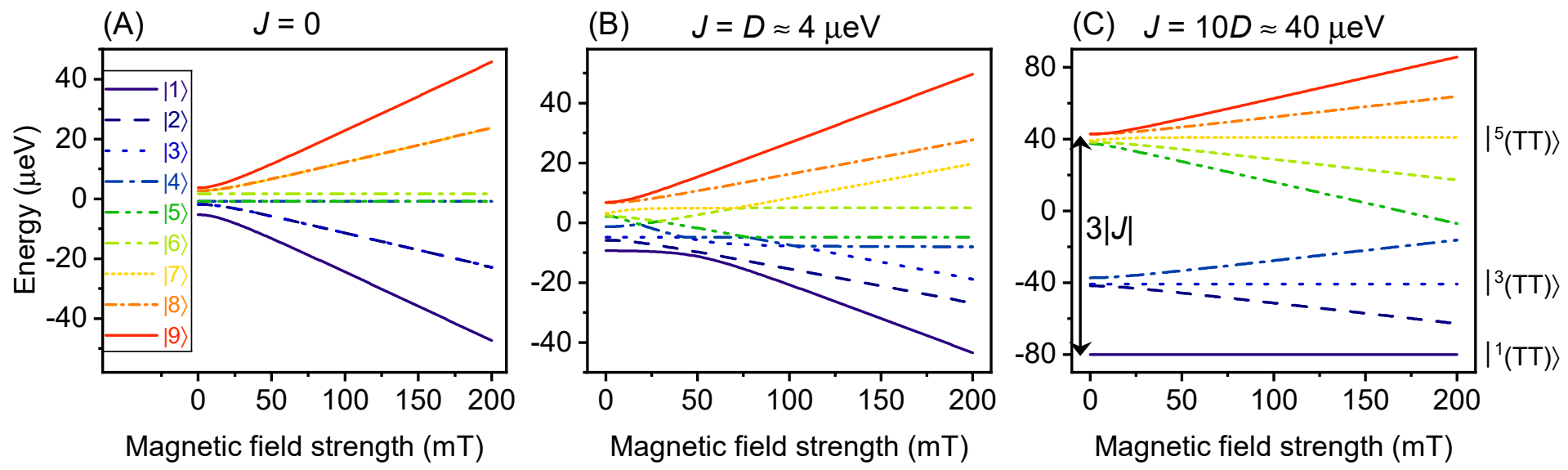

Fig. S3: (A) When $J = 0$, the states are energetically close as defined by the D and E parameters. Importantly, many of the states will have near-degeneracies. (B) When $|J| = |D|$, the states are further apart, and interactions occur at higher fields when compared to the $J = 0$ case. (C) When $|J| = 10|D|$, the states are spaced much further apart, and the limited interactions occur only at avoided level crossings. The lowest lying state, $|1\rangle$, has pure singlet character.

When $J = 0$ eV (Fig. S3a), the nine states are energetically closely spaced at zero field, with the small energetic splitting defined by the D and E parameters. In this case, many of the states exhibit near-degeneracies, and spin is not a good quantum number (i.e., it is not possible to assign a unique spin quantum number to these eigenstates).

In contrast, when $|J| \gg |D|$ (Fig. S3c), the nine states are energetically separated according to their well-defined spin quantum numbers: one singlet $^1(T_1T_1)$ with spin quantum number $S = 0$, three triplets $^3(T_1T_1)$ with $S = 1$, and five quintets $^5(T_1T_1)$ with $S = 2$. The energetic splitting between $^1(T_1T_1)$ and $^5(T_1T_1)$ is $3|J|$.

Before continuing we pause to clarify the notation we're using. Following recent convention in the literature [5], when it is useful to differentiate between limiting cases, we denote the strongly exchange-coupled ($|J| \gg |D|$) triplet pair as (TT) and the weakly exchange-coupled triplet pair ($J \approx 0$) as (T··T). This notation is not universal and in earlier works, such as the original Merrifield Johnson work [6, 7], the term ^{1}(TT) was used to denote weakly exchange-coupled triplet pairs. In the following we distinguish strongly exchange coupled (TT) from weakly exchange coupled (T··T) when the limiting cases are important. We use the former notation when describing the generic case, i.e. when describing the spin Hamiltonian with varying exchange, J.

Magnetic Field Dependent Singlet Fission Probability

Singlet fission is a spin-conserving process, and its rate depends on the 'singlet character' of the triplet-pair states. The singlet character, in turn, is affected by the applied magnetic field.

Singlet character refers to the degree to which a triplet-pair state has the same spin symmetry as a singlet state. Mathematically, it is quantified by $C_S^l = \langle \psi_S | (\mathrm{T_1T_1})^l \rangle$, the inner product, or overlap, between the singlet wavefunction $|\psi_S\rangle$ and $|(\mathrm{T_1T_1})^l\rangle$ (the triplet-pair eigenstates of the spin Hamiltonian described above). The *probability* ($P_{c_S^l}$) of transitioning from the singlet state to the $(\mathrm{T_1T_1})^l$ state is calculated by computing the squared modulus of C_S^l:

$$P_{c_S^l} = |C_S^l|^2 = |\langle \psi_S | (\mathrm{T_1T_1})^l \rangle|^2 \tag{3}$$

The rates of SF, k_{SF}, and its reverse, TTA, k_{invSF} are modulated by this probability, such that:

$$\mathrm{S_0S_1} \underset{k_{\mathrm{invSF}}|C_S^l|^2}{\overset{k_{\mathrm{SF}}|C_S^l|^2}{\rightleftharpoons}} (\mathrm{T_1T_1})^l \tag{4}$$

We can now consider how the magnetic field alters the overall rate of SF in different limiting cases based on the relative magnitude of the exchange interaction ($|J|$) between the triplets in the pair: either $J = 0$ (Fig. S3a), or $|J| \gg |D|$ (Fig. S3c).

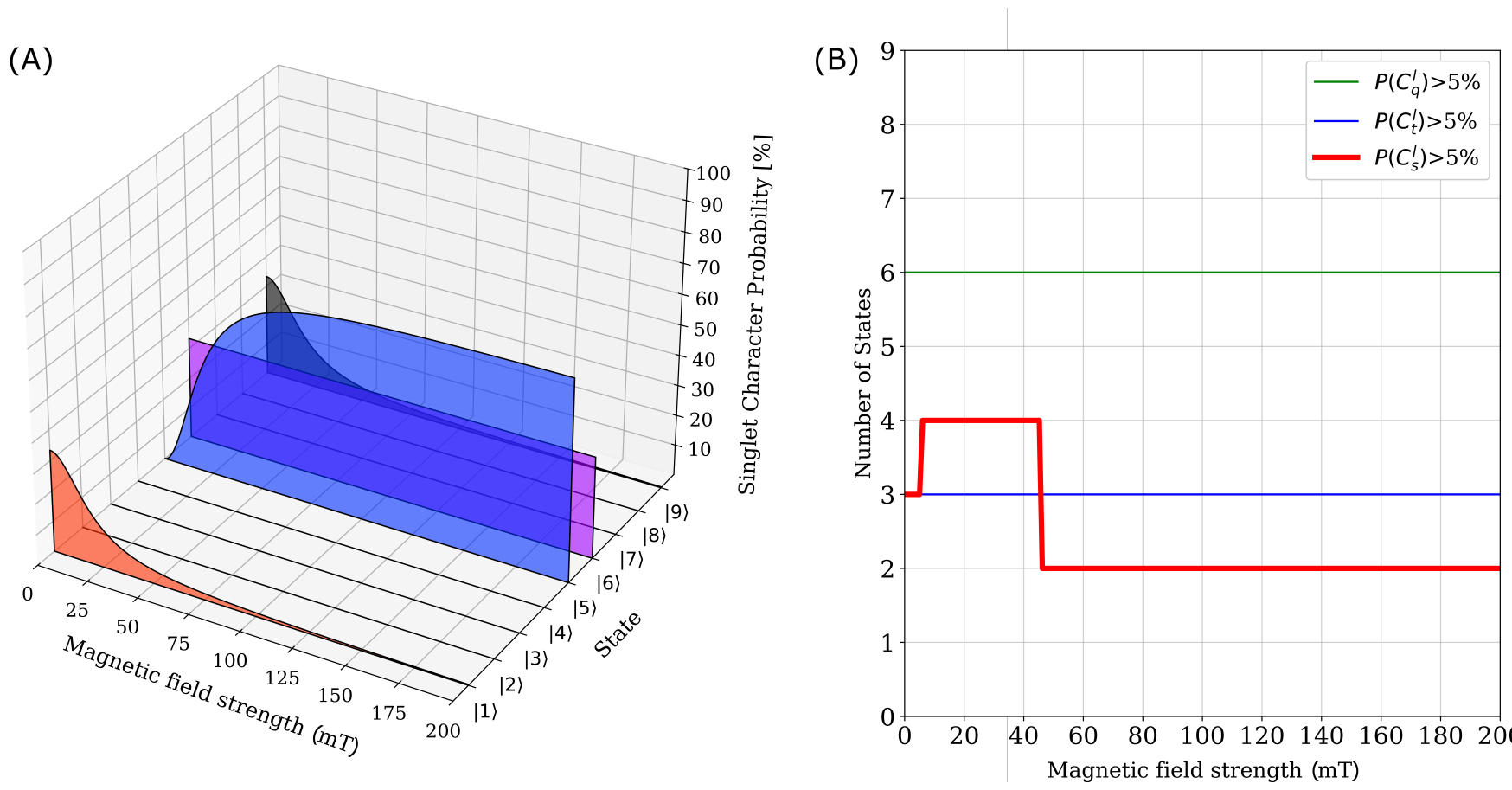

Fig. S4: (A) The changes in singlet character probability by state. Starting with three states at 33 % probability at zero field, four states assume singlet character at low fields and two states remain with singlet character at high fields. (B) An arbitrary threshold of 5% is selected to visualise the number of states with singlet, triplet and quintet character.

For $J = 0$, the number of $(\mathrm{T_1 \cdot\cdot T_1})^l$ states with singlet character changes with applied magnetic field. Fig. S4a plots the singlet character probability ($P_{c_S^l} = |C_S^l|^2$) of

the nine triplet-pair states as a function of magnetic field. Fig. S4b shows the number of states with more than 5 % probability of singlet (red), triplet (blue) or quintet (green) character as a function of magnetic field strength.

At zero field, three states have significant singlet character. As the field increases to $|B| \approx |D|/g\mu_B$, a fourth state gains singlet character due to increased mixing between states. At still higher fields $|B| > |D|/g\mu_B$, the mixing is reduced, and only two states retain singlet character. Since the SF rate is proportional to the number of states with singlet character, it increases in the first regime and decreases in the second.

For $|J| \gg |D|$, the number of $(\mathrm{T}_1\mathrm{T}_1)^l$ states with singlet character *does not change* with applied magnetic field, up to $|B| = |J|/2g\mu_B$, where level crossings between singlet and quintet triplet-pair states occur. For $|J| = 10|D| \approx 40$ μeV, this crossing is expected near $|B| \approx 550$ mT, which lies outside our measurement range. Therefore, with $|J| \gg |D|$, the rate of SF will be independent of magnetic field strength in our measurement range.

Magnetic Field Dependent Photoluminescence

The kinetic model for MFE simulations

To interpret our MFE results, we constructed a simplified kinetic model, shown schematically in Fig. S5. The model includes singlet fission (SF) from S_2, modulated by the spin Hamiltonian ($k_{SF}|C_S^l|^2$, with $k_{SF} = 1/1.2\,\text{ps}^{-1}$), energy transfer from S_2 to Q_y ($k_{ET} = 1/0.17\,\text{ps}^{-1}$) (this is an effective rate, including all purely singlet exciton energy transfer pathways *via* S_1 or Q_x), triplet-triplet annihilation (TTA) from the triplet pair states to Q_y (again modulated by the spin Hamiltonian, $k_{invSF}|C_S^l|^2$ with $k_{invSF} = 1/10^5\,\text{ps}^{-1}$) and radiative/non-radiative decay to the ground state ($k_n rad = 1/10^3\,\text{ps}^{-1}$). The rates were estimated from the time-resolved spectroscopy in the main text and the D and E parameters from magnetic resonance studies in the literature [4]. We assume the triplets within the pair are oriented parallel to each other. Fig. S5B shows the resulting simulated population of the Q_y state with $J = 0$ as a function of time, with zero, 15 mT and 200 mT applied magnetic field.

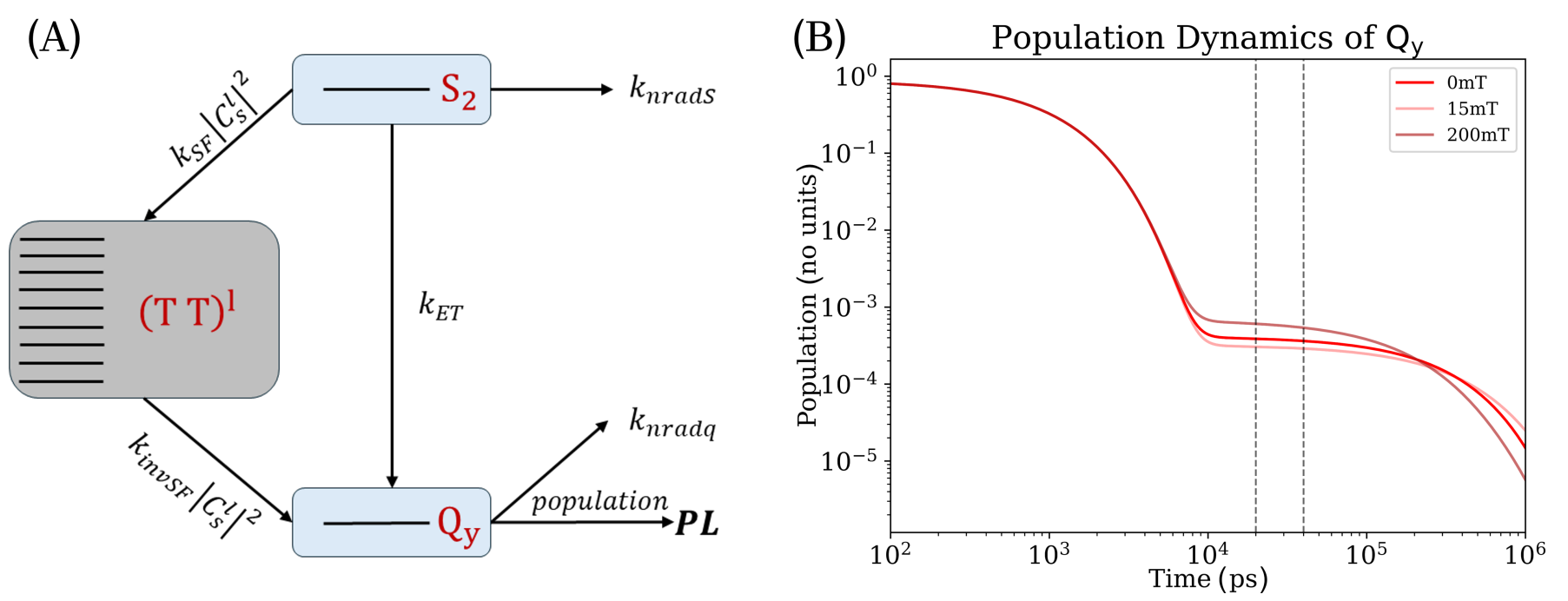

Fig. S5: (A) Simplified schematic of the kinetic model with S_2, $(T_1 \cdot\cdot T_1)^l$ and Q_y transitions outlined. We assume the population of Q_y to represent the PL. (B) Simulated dynamics of the lowest lying singlet state at 0 mT, 15 mT and 200 mT. The dotted lines represent 20 ns and 40 ns time window of the experimental MFE measurements.

Photoluminescence lineshape and its magnetic field dependence

To compare directly with our experimental results in Fig. 2 of the main text, where we plotted the relative change in BChl *a* emission as a function of magnetic field ($\Delta PL(B)/PL$), measured at 880 nm within a $20 - 40$ ns delay window, we focus on the same time window (20-40 ns) in the simulated data. This is marked by the dashed lines in Fig. S5B. In this time window, we observe the familiar SF MFE: a decrease in PL at low fields (15 mT) compared with zero-field, followed by an increase at higher fields (200 mT).

Fig. S6A extends the simulation to a larger range of magnetic fields, plotting the change in Q_y population at 20-40 ns after excitation compared with the zero-field case

for direct comparison with the experimentally determined $\Delta PL/PL$. Panel A of the figure shows the MFE curves for $J = 0$, $|J| = |D|$ and $|J| = 10|D|$. As shown in Fig. S6B, only the weak coupling case (i.e., $|J| \ll |D|$) reproduces the experimental ΔPL/PL response for Spn-RC–LH1.

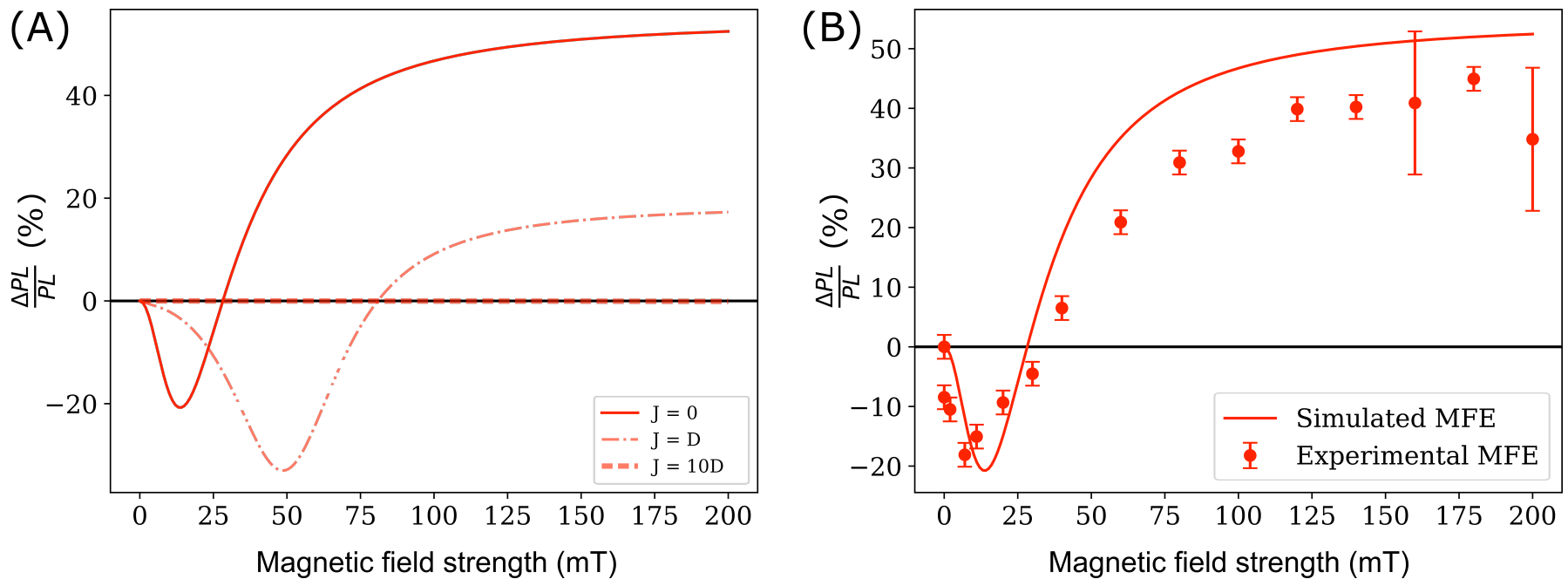

Fig. S6: (A) Simulated magnetic field effects of the Q_y population as a function of applied field PL changes (BChl *a* Q_y) using the kinetic model described above at 20-40 ns after excitation under three exchange coupling scenarios: J is set to zero, equal to the D tensor, and ten times the magnitude of the D tensor. Only the weak coupling case ($J \ll D$) reproduces the experimental field dependence. (B) Direct comparison between the experimental MFE of Spn–RC–LH1 in Fig. 2 of the main text (measured at 20-40 ns after excitation) and the simulated case under weak coupling conditions (also 20-40 ns after excitation). Error bars: 2σ (standard deviation) from subsequent field up and down scans.

Here, for completeness, we describe the MFE shapes in more detail. When $J = 0$, the PL intensity observed at zero field reflects a baseline SF rate, where three of the nine triplet-pair states possess significant singlet character. Upon application of a low magnetic field ($10 - 40$ mT), a fourth state acquires singlet character, enhancing the SF rate. This leads to a reduction in PL intensity, seen as a negative ΔPL/PL signal. At higher fields, two states lose singlet character, reducing the SF rate and causing the PL intensity to rise again. Thus, the magnetic field modulates the likelihood of SF by changing the singlet character of the triplet-pair states. As a result, PL becomes a sensitive probe of spin-state mixing in the system.

When $|J| \gg |D|$, the singlet and quintet states are energetically well-separated by $\sim 3|J|$, see Fig. S3, and spin-state mixing is suppressed. The singlet character of the triplet-pair states is fixed, rendering the SF rate and PL intensity independent of magnetic field within the experimental range ($B < 200$ mT). However, reductions in PL intensity may occur at higher magnetic fields corresponding to level anticrossings between singlet and quintet states [3, 8]. In these regions, the Zeeman interaction can partially cancel the exchange interaction.

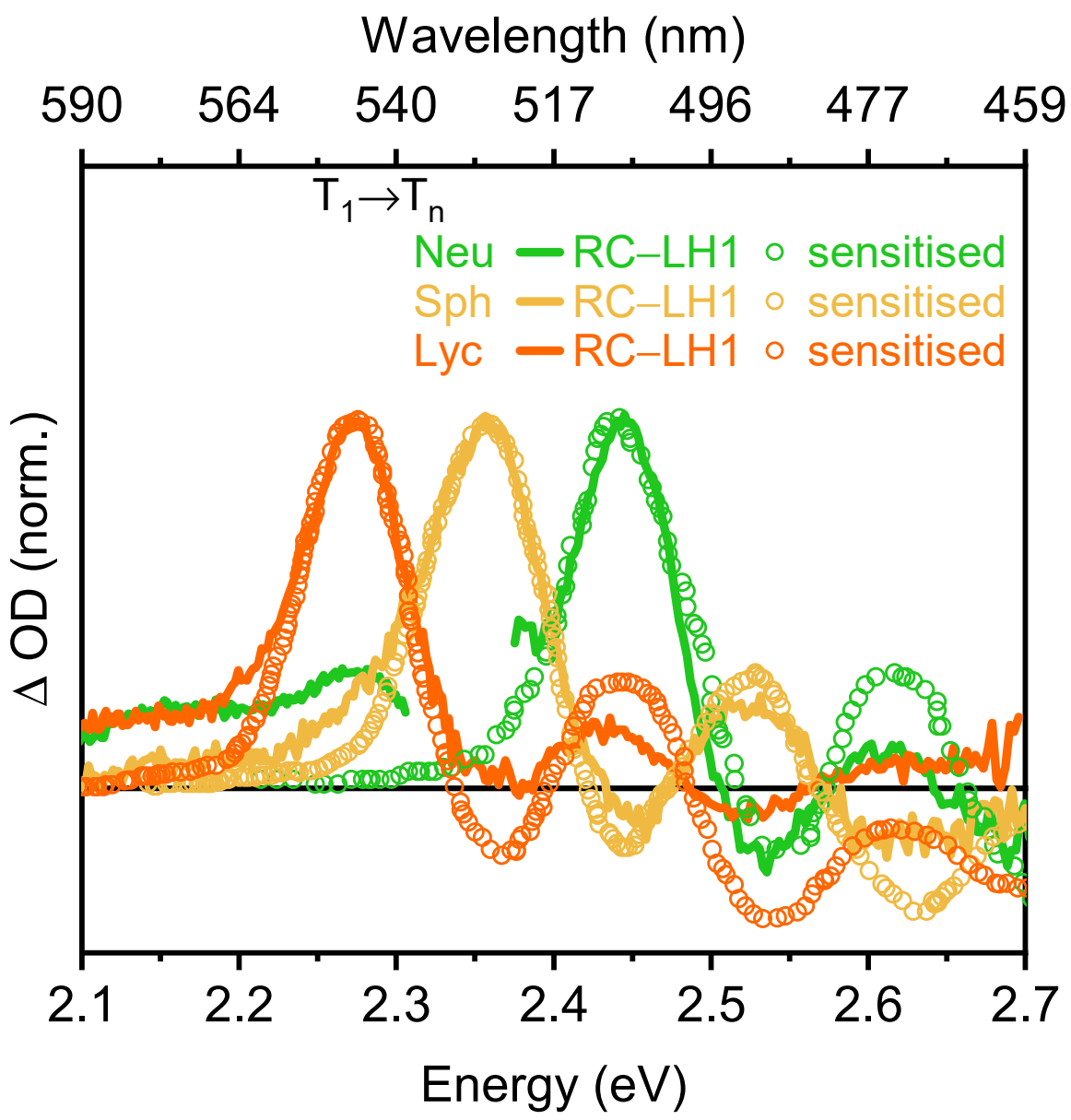

Fig. S7: Transient absorption spectra of the RC–LH1 complexes containing neurosporene (Neu), spheroidene (Sph), and lycopene (Lyc) (solid, $\Delta t = 5\,\mu s$) were measured by nanosecond transient absorption spectroscopy. Carotenoid excited triplet state spectra were obtained at $1.8\,\mu s$ after triplet-sensitised excitation (circles), extracted from Ref. [9] (used with permission).

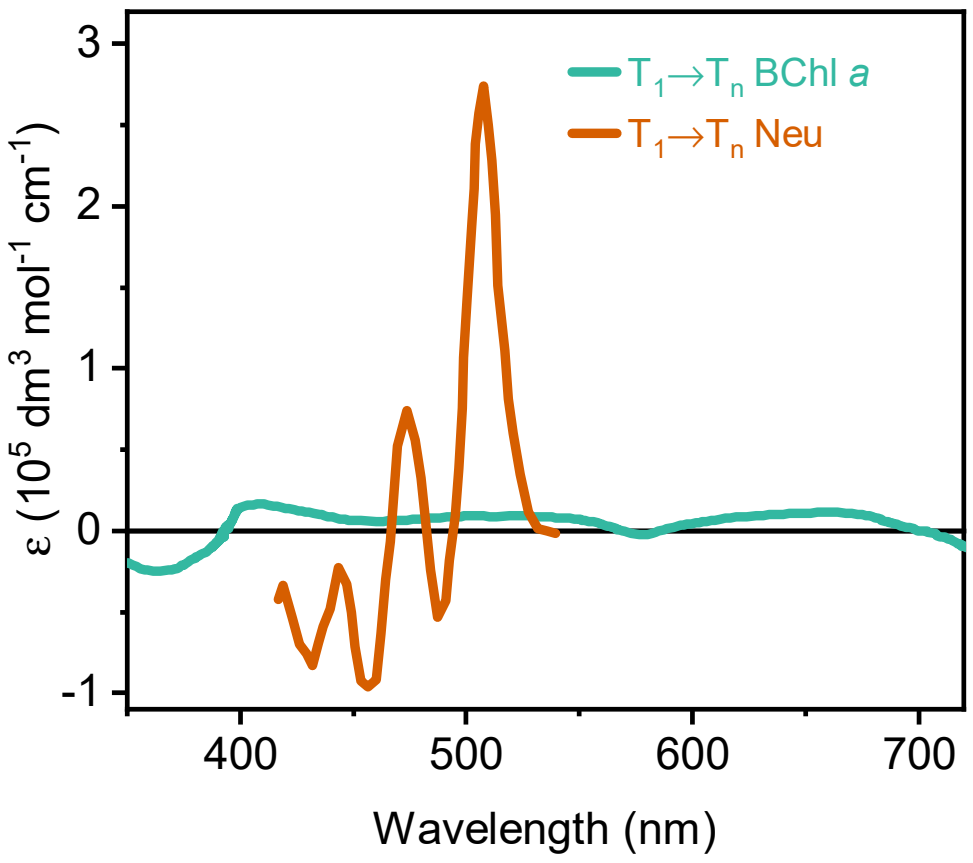

Fig. S8: Extinction spectra of BChl *a* $T_1 \rightarrow T_n$ excited state absorption reproduced from Ref. [10] (used with permission), and Neu $T_1 \rightarrow T_n$ excited state absorption spectrum extracted from Ref. [9] (used withe permission), scaled by the extinction coefficient inRef. [11]. As observed in the figure, Crt molecules have relatively large excited state extinction coefficients for triplets compared with those of BChl *a* – estimated to be roughly ten times larger ($\sim 2 \times 10^5\,\mathrm{M^{-1}\,cm^{-1}}$ [11]) than the BChl *a* peak triplet absorption coefficient ($\sim 0.2 \times 10^5\,\mathrm{M^{-1}\,cm^{-1}}$ [10]). The transient absorption signals of Crt–RC–LH1 in the visible spectral region are therefore dominated by Crt excited state features and the signatures of BChl *a* are obscured, making assignment difficult.

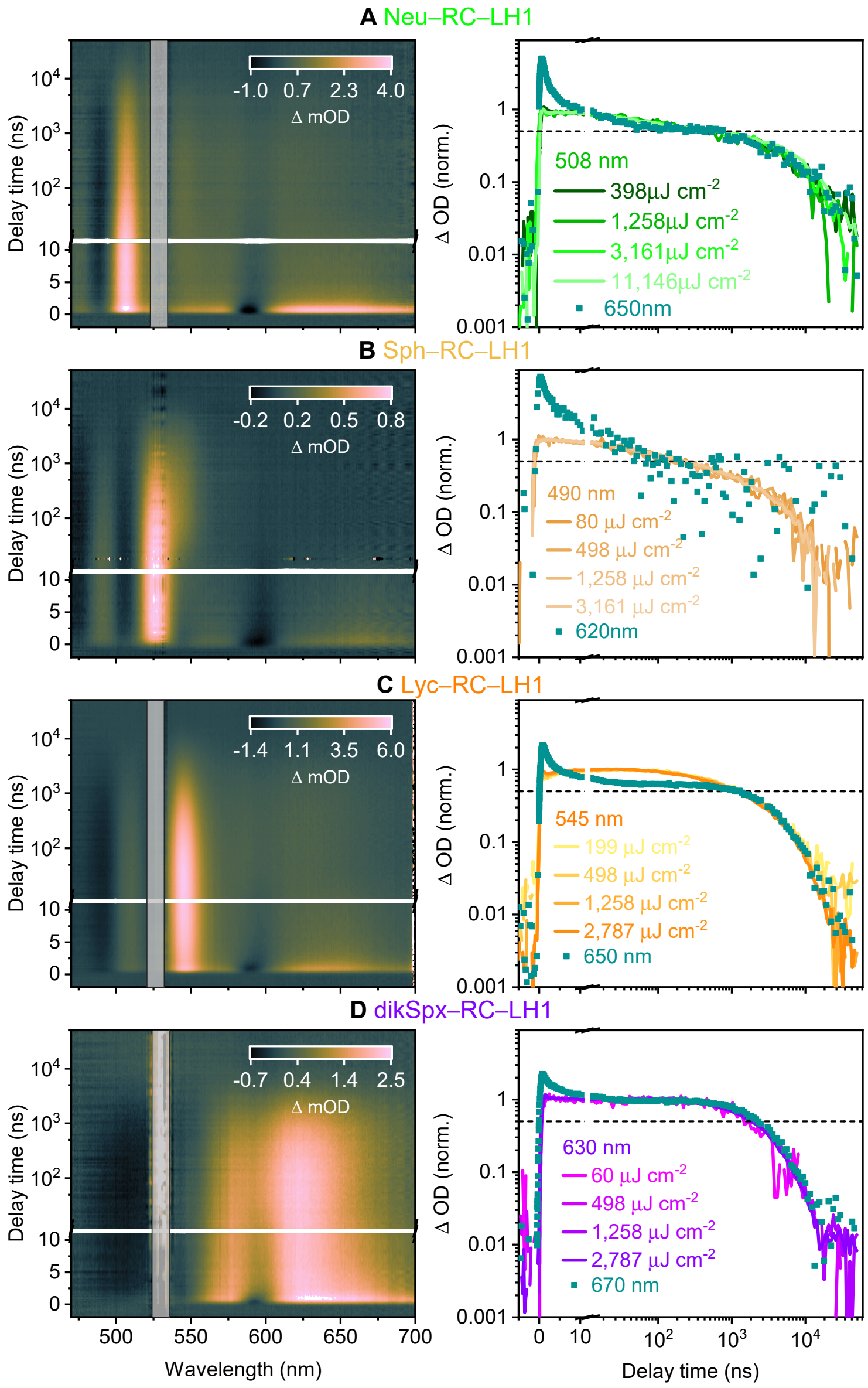

Fig. S9: 2D transient absorption maps (left panels) and normalised kinetic profiles (right panels) for RC–LH1 complexes containing (**A**) neurosporene (Neu), (**B**) spheroidene (Sph), (**C**) lycopene (Lyc), and (**D**) 2,2'-diketo-spirilloxanthin (dikSpx) upon excitation at 532 nm (500 ps pulses, ∼2 ns IRF) - *caption continued on next page...*

Figure S9 (continued)
Right panels: kinetic profiles at different laser fluences (marked in the legend) within the Crt triplet ESA bands at probe wavelengths 508 nm (Neu), 490 nm (Sph), 545 nm (Lyc), and 630 nm (dikSpx) and within the much weaker BChl *a* ESA band (between $620-680$ nm, teal). Dashed horizontal lines indicate point at which half the Crt triplet population has decayed. The data were acquired up to $\Delta t = 50\,\mu s$, using a linear time scale up to 11 ns and a logarithmic time scale from $11-50000$ ns. 532 nm excites both Crt and BChl *a*, and has weak absorption in the Neu–RC–LH1 sample. Each TA map features a prominent long-lived ($\sim$ μs) excited state absorption (ESA) band associated with the Crt triplet and weaker signatures of BChl *a* GSB (~ 600 nm) and ESA across the visible range. The time profiles of carotenoid $T_1 \rightarrow T_n$ ESA bands, lines in the right panels show no excitation density dependence for any of the complexes, indicating that bimolecular processes are not occurring. The Neu and Sph triplets (lines in panels A, B) start to decay from the first few nanoseconds and show non-exponential decay across the entire time range with a half-life (indicated by the dashed lines) of $\sim 0.3-0.4\,\mu$s. Triplet dynamics in Lyc–RC–LH1 complexes (panel C, orange/yellow lines, $\lambda_{\mathrm{probe}} = 545$ nm) show a delayed *rise* over the first few tens of nanoseconds, most likely due to triplet energy transfer from BChl *a* triplets (generated by intersystem crossing). In dikSpx–RC–LH1 complexes (panel D, purple lines), a less prominent rise is apparent. In (C) and (D), the Crt triplets decay more slowly compared with Neu- and Spe-triplets, with half-lives of $\sim 1-3\,\mu$s.

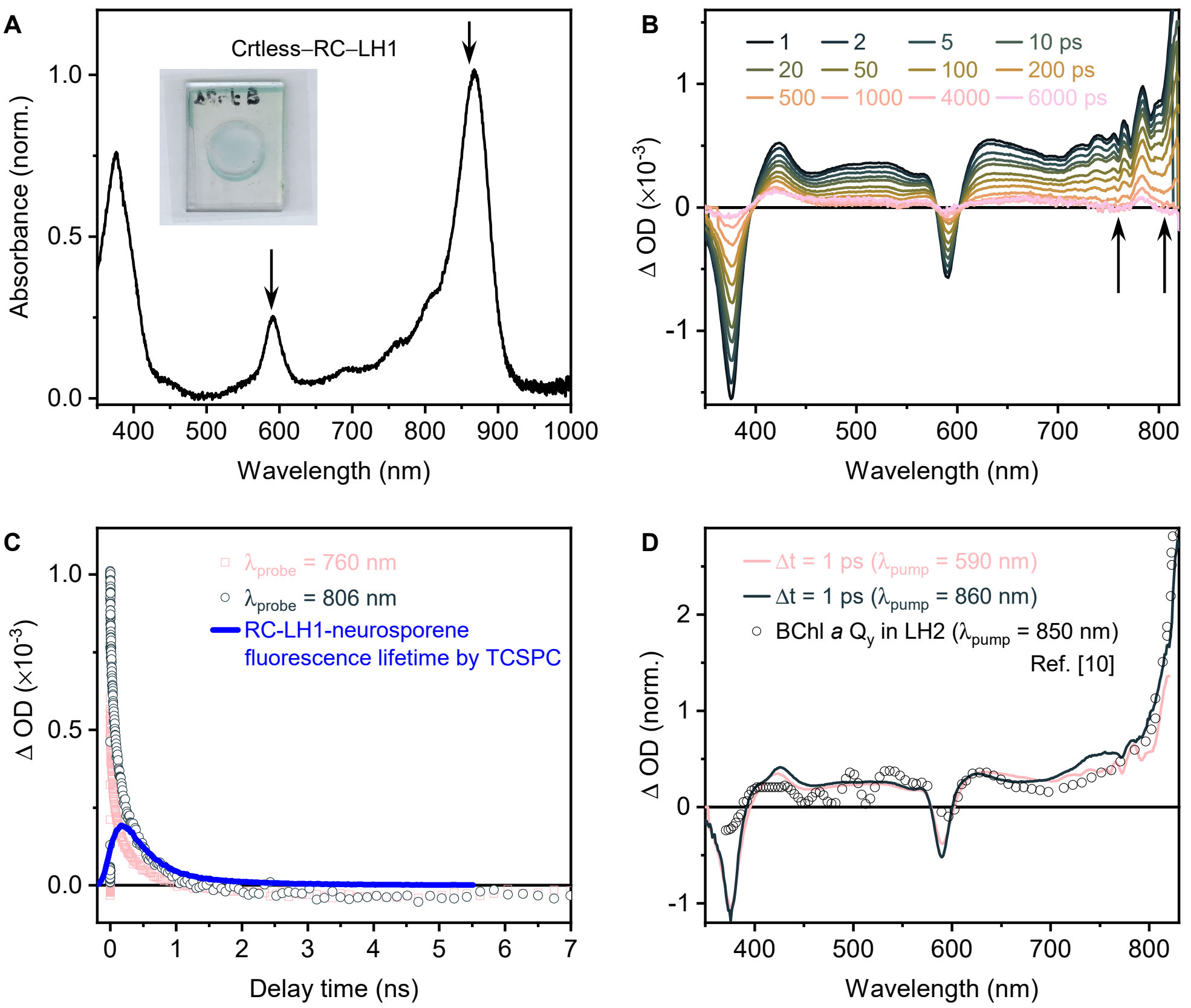

Fig. S10: Crtless-RC–LH1 absorption spectroscopy: (**A**) Static absorption spectrum of the Crtless–RC–LH1 complex. Excitation wavelengths (590 and 860 nm) are indicated by vertical arrows. Inset: photograph of the sugar film. (**B**) Transient absorption spectra of the Crtless–RC–LH1 complex after BChl *a* Q_x excitation at 590 nm for delay times between 1 − 6000 ps. Vertical arrows indicate the selected probe wavelengths (760 and 806 nm) for plotting the corresponding dynamics in panel (C). During the entire time window, the spectral features change only slightly. However, further inspection demonstrates differences in the region between 750 − 850 nm as a function of time. In this spectral region, the positive excited state absorption (ESA) at early times becomes negative ground state bleach (GSB) at late times. (**C**) Dynamics at selected wavelengths 760 and 806 nm decaying to negative after 1 ns and the presence of a long-lived (non-decaying) bleach feature at these wavelengths. Also plotted is the fluorescence decay (900 nm) measured with TCSPC, normalised to 0.7 ns. The early-time decay transient absorption decay (< 1 ns, ESA signal) matches with the BChl *a* Q_y fluorescence lifetime showing that the positive excited state absorption (ESA) band between 750 − 850 nm must be associated with Q_y population. Note that TCSPC has a lower temporal resolution which obscures the early-time (sub-200 ps) decay. (**D**) BChl *a* Q_y spectrum obtained at early time (Δt = 1 ps) following Q_x (590 nm) and Q_y (860 nm) excitation and the reported BChl *a* Q_y excited state spectrum measured in LH2 complex from *Rba. sphaeroides*, from Ref. [12]. We assign the ESA band between 750 − 850 nm to $Q_y \rightarrow S_n$ absorption. In RC–LH1 Q_y decays with a ∼ 1 ns time constant.

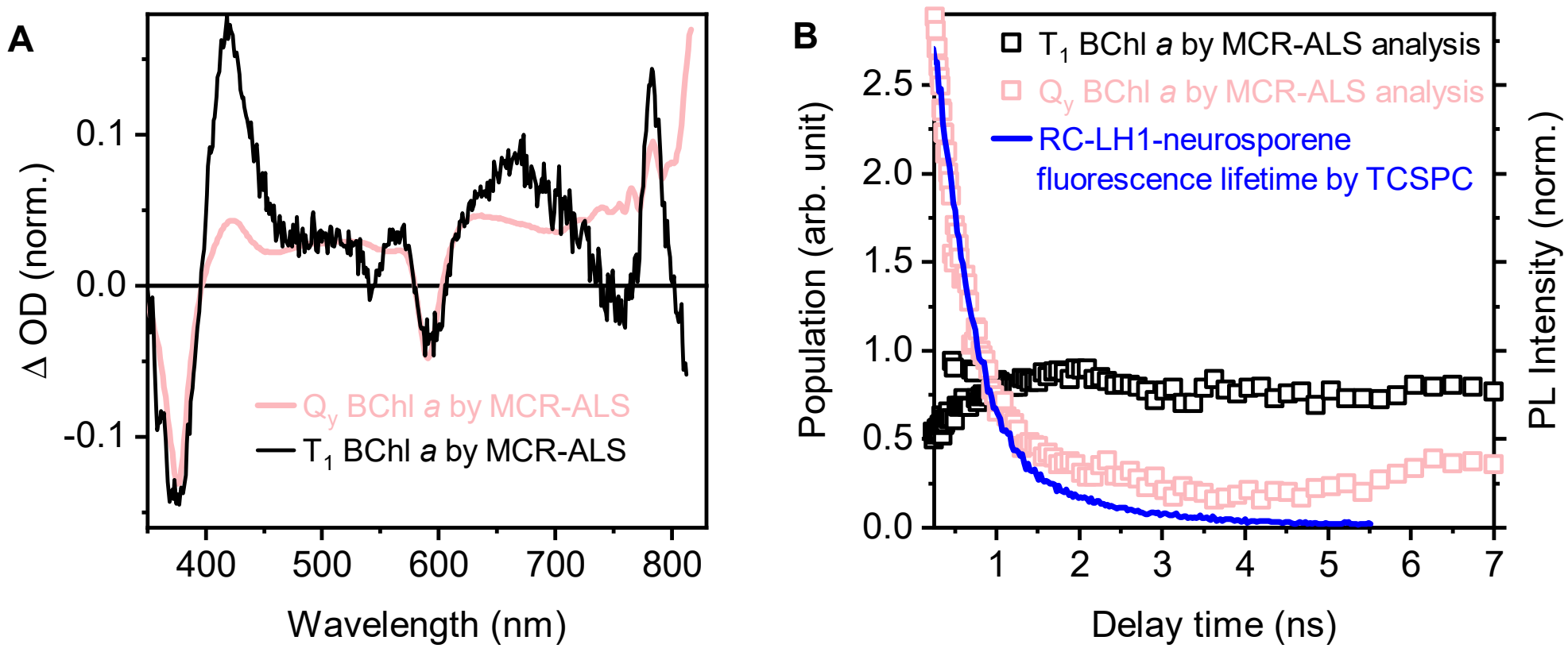

Fig. S11: Global Analysis using multivariate curve resolution-alternating least squares (MCR–ALS see Refs. [13, 14]) of Crtless–RC–LH1 from Fig. S10 after excitation into the BChl *a* Q_x band. Two extracted components with corresponding spectra (**A**) and dynamics (**B**) in black and pink. We assign the pink component to Q_y as it closely matches the spectra reported in Fig. S10D and because its population decay is similar to the measured Q_y fluorescence (blue curve in panel B). As the black spectral component rises as the Q_y feature decays, barely decays over $1-7$ ns and has spectral features of BChl *a*, we assign it to the triplet T_1 excited state spectrum. This assignment is supported by the spectral similarity to the flash photolysis (μs) excited state spectrum of a Crtless mutant *Rba. sphaeroides* chromatophore reported in Ref. [15] and the T_1 spectrum of BChl *a* in pyridine, measured with flash photolysis in Ref. [16] (in RC–LH1, the Q_y GSB feature is red-shifted compared with the solution) and the absorption-detected microwave resonance (ADMR) spectra of triplets in the reaction centre from *Rba. sphaeroides* [17].

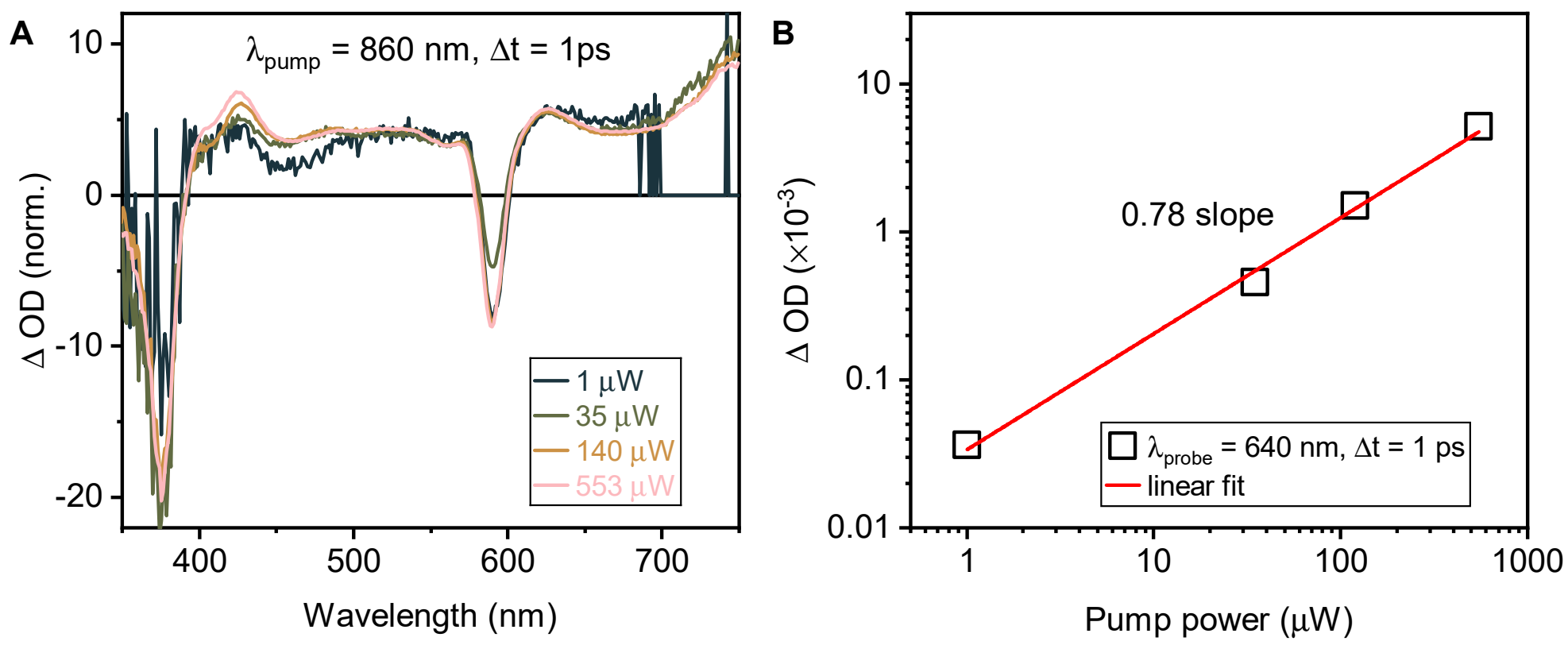

Fig. S12: Pump power dependence of Crtsless–RC–LH1 complex from *Rba. sphaeroides*. Porphyrins have been reported to demonstrate efficient step-wise two-photon absorption through the S_1 state (the Q state) [18], even with relatively low power densities [18]. (**A**) shows normalised transient absorption spectra of the Crtless–RC–LH1 complex from *Rba. sphaeroides* for delay time of 1 ps after BChl *a* Q_y excitation at 860 nm with pump power from $1 - 553\,\mu$W (200 μm diameter pump spot, 5 kHz rep. rate). (**B**) shows dependence of the ESA signal at 640 nm on the pump pulse power. The red line in the log-log plot is the best fit of the experimental data (squares) and has a slope of 0.78. BChl *a* in RC–LH1 therefore demonstrates weak non-linear behaviour at fluences typical of our measurements likely associated with two-photon or sequential excitation to form states other than Q_y. Determining the reason for the spectral shape change at increased fluence is beyond the scope of this work. However, this non-linear behaviour (two photons generate a single excited state) means that triplet yield measurements [19] which use pulsed lasers should ensure that non-linear effects such as this are controlled for.

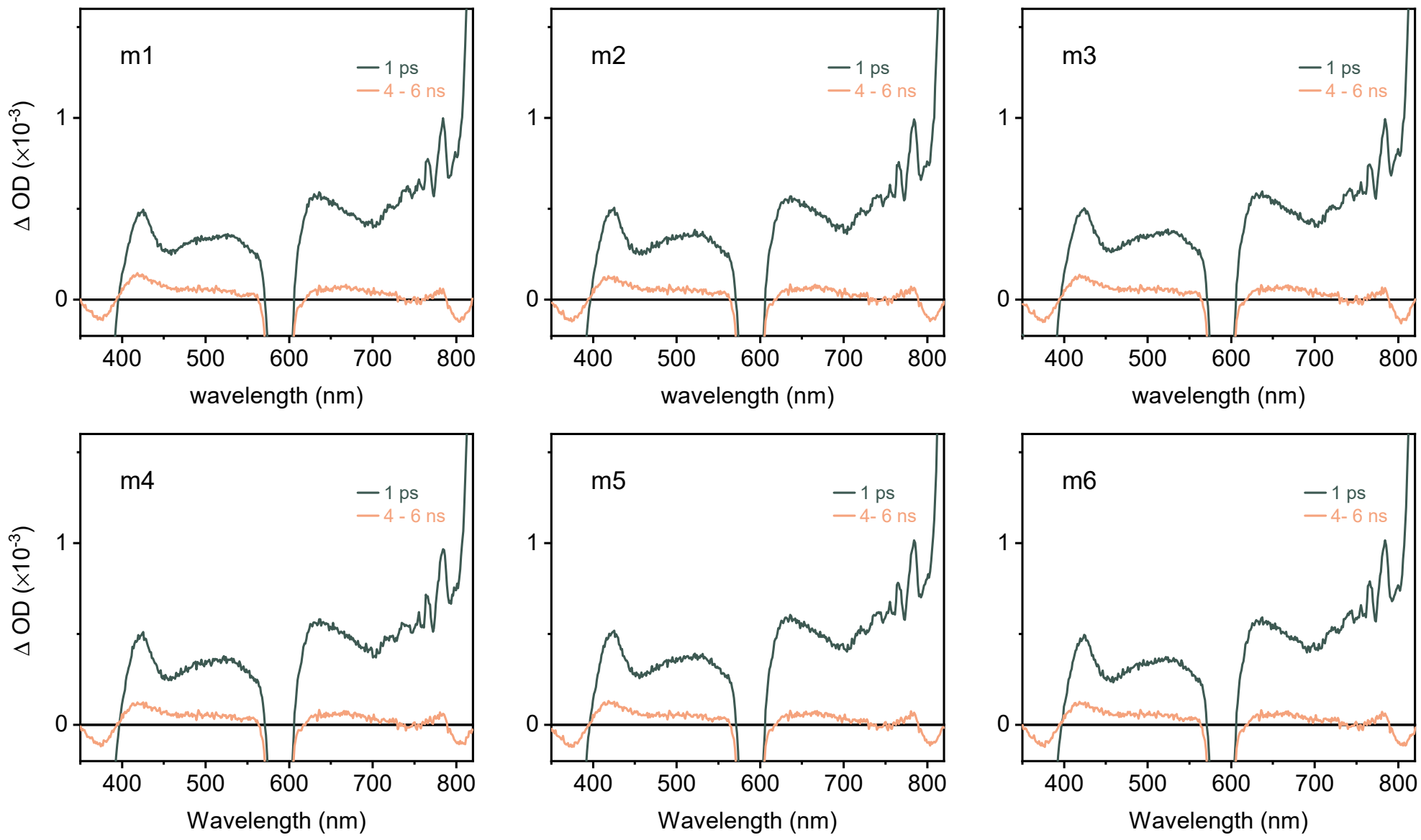

Fig. S13: Fig. 3C in the main text and Fig. S10B show the averaged results from 6 independent measurements (each consisting of ~ 10 scans). To ensure the robustness of the observed spectral features, all 6 individual datasets are also shown here for comparison.

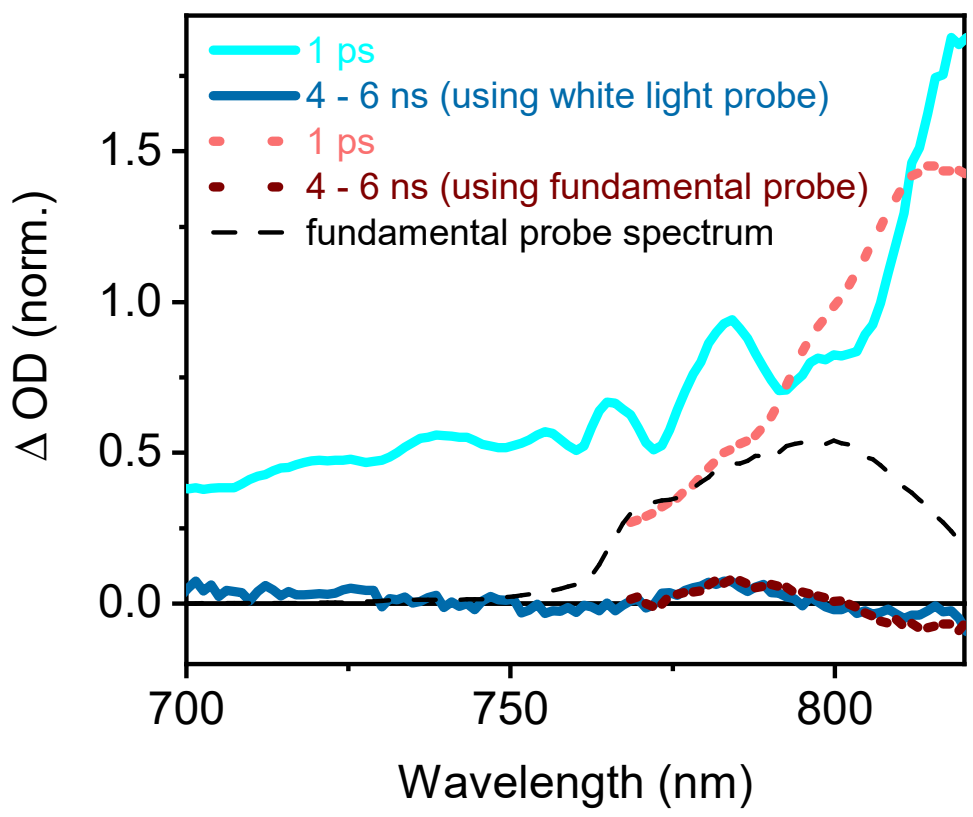

Fig. S14: We performed multiple measurements on the Crtless sample following 590 nm excitation, using different probe sources to validate the observed signals in the 750–820 nm region. Consistently, we detected spectral signatures corresponding to both the BChl *a* singlet and triplet states. To probe this region of interest, we employed a white-light supercontinuum spanning $350 - 820$ nm, as well as the 800 nm fundamental (indicated by the black dashed line). Both probes revealed the same spectral features: a positive absorption signal for the BChl *a* Q_y transition and a weak negative bleach signal corresponding to the T_1 state.

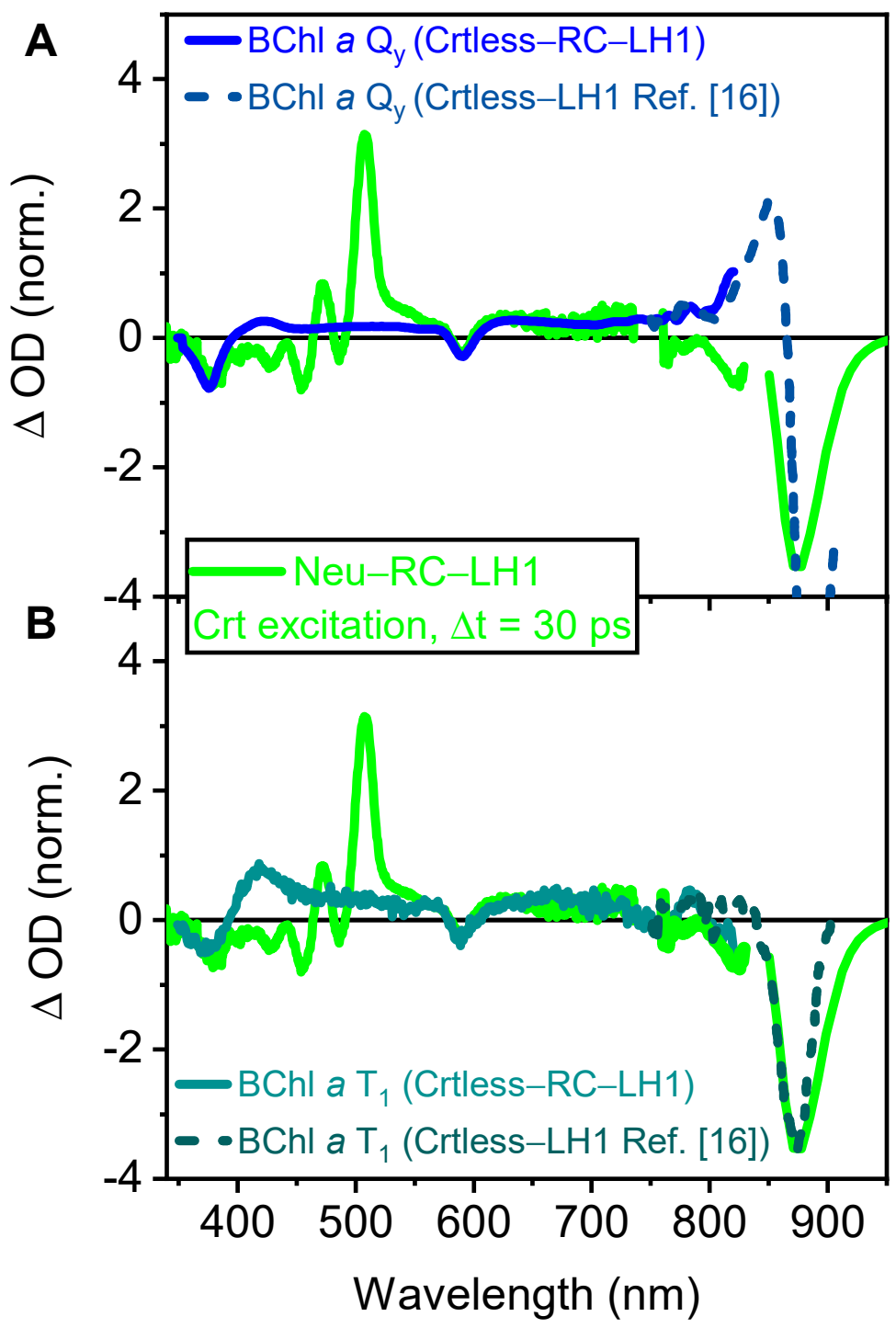

Fig. S15: Assignment of Neu–RC–LH1 BChl *a* spectral features at 30 ps following Neu S_2 excitation by comparison with Q_y (**A**) and T_1 (**B**) reference spectra. Solid-line reference spectra are from *Rba. sphaeroides* Crtless–RC–LH1 (see Fig. S11) and dahsed-line reference spectra from Ref. [20] (from Crtless–LH1 complexes from *Rsp. rubrum*, known to show very similar protein/pigment structure and photophysics compared to LH1 complexes studied here). The spectra from Ref. [20] (used with permission) overlap well with the spectra reported here between 750 − 850 nm. The comparison between Neu–RC–LH1 at 30 ps following Neu S_2 excitation (pale green) and the Q_y and T_1 reference spectra suggest that Neu S_2 excitation generates BChl *a* T_1 states by 30 ps, rather than only Q_y states.

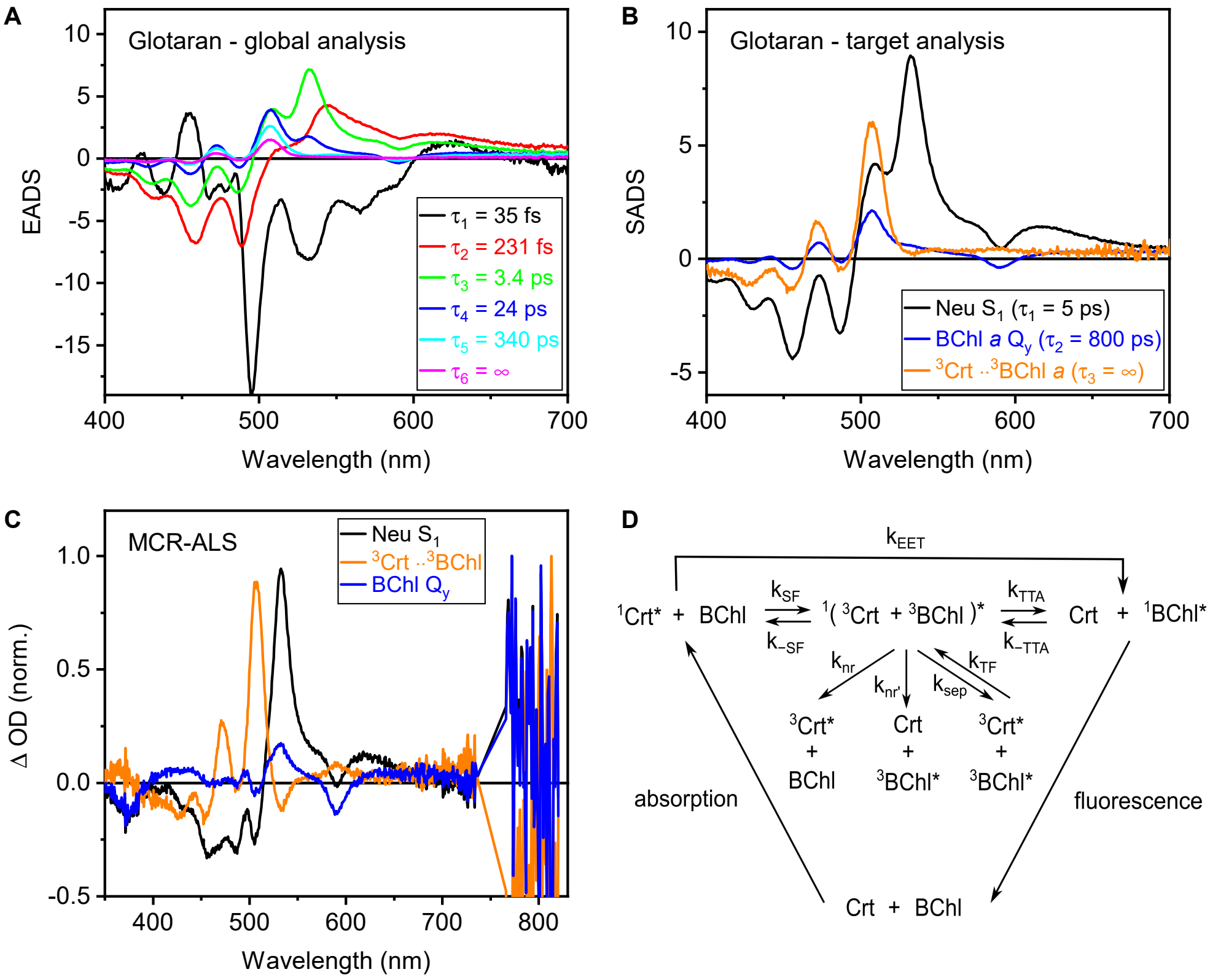

Fig. S16: Global analysis of transient absorption data of Neu–RC–LH1 with Neu S_2 excitation does not result in pure species. We used both Glotaran [21] (A-B) and MCR-ALS [13, 14] (C) global analysis. (**A**) Evolution-associated absorption difference spectra (EADS) of photoexcited Neu–RC–LH1 obtained *via* singular value decomposition-based Glotaran global analysis [21] using six components and a dataset of $400 - 700\,\text{nm}$, $-0.5 - 7000\,\text{ps}$ for comparison with literature [22]. (**B**) Species-associated absorption difference spectra (SADS) obtained by Glotaran target analysis using a kinetic model, see (D). The spectra and time constants are in good agreement with those in Ref. [22]. However, the species cannot be assigned to pure excited state populations. For example, the species with a time constant of $\sim 5\,\text{ps}$, assigned to Neu S_1 also contains the contributions from Neu T_1 and BChl *a* states. (**C**) Species spectra extracted by MCR–ALS global analysis. The dataset of $350 - 820\,\text{nm}$, $1 - 7000\,\text{ps}$ was used to simplify the photophysics of excluding of Neu S_2 and BChl *a* Q_x states. Unfortunately, we observe clear correlations between spectra (blue and orange spectra are anti-correlated, the Neu-S_1-assigned spectrum shows BChl *a* GSB signatures). The algorithm cannot therefore successfully separate the states, perhaps because the BChl *a* spectra are too similar or their signals are too weak (compared to the Neu transient signals). We ran the algorithm with different starting conditions, different numbers of states and different data sets, but could find no combination that generated uncorrelated spectra. (**D**) Simplified kinetic scheme following Crt excitation, rates (k_i) are defined by this scheme

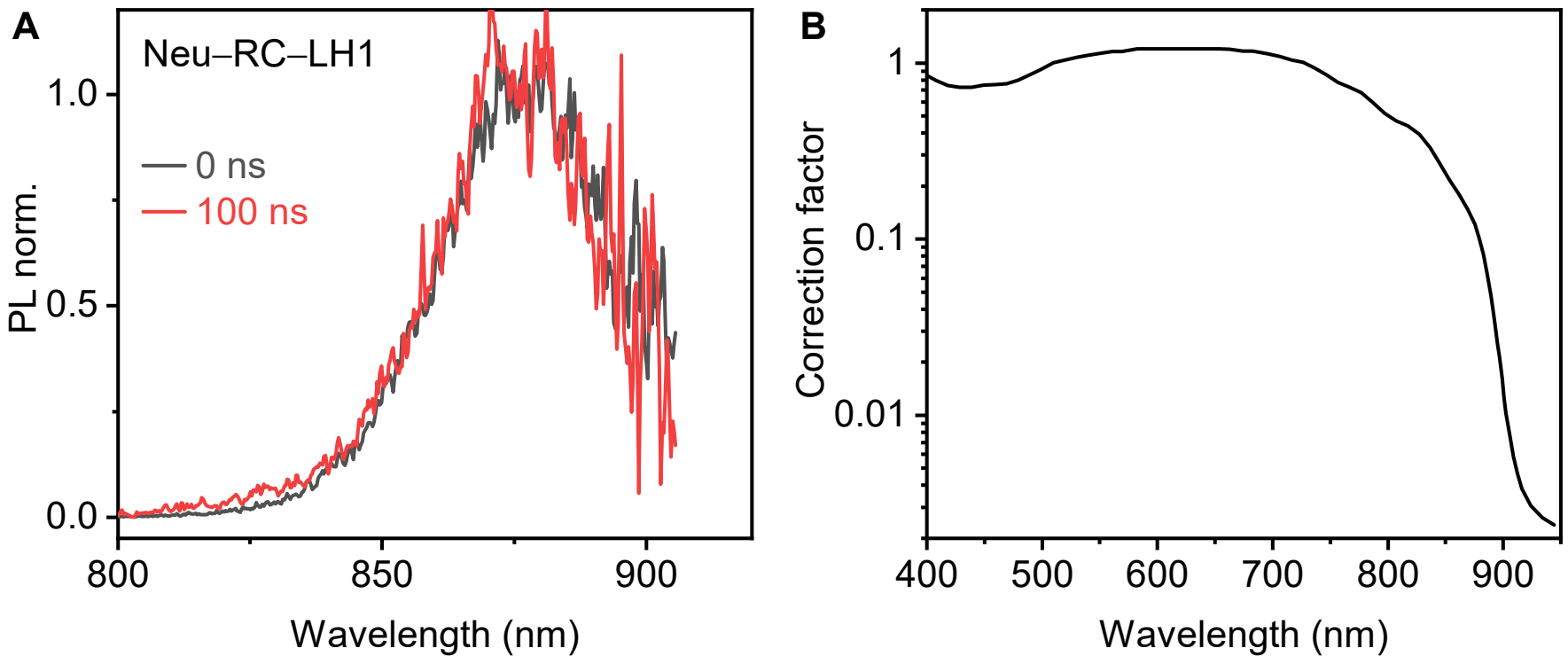

Fig. S17: (**A**) Prompt (black) and delayed (red) emission spectrum of the Neu–RC–LH1 complex at delay time $\Delta t = 0$ ns (within 4 ns of excitation due to pulse jitter) and $\Delta t = 100$ ns, respectively. Excitation at 500 nm. The spectral shape does not change over time, i.e., the delayed emission spectrum (at 100 ns) of Neu–RC–LH1 after Neu excitation at 500 nm is the same as the fluorescence spectrum immediately after excitation, and is assigned to BChl *a* Q_y fluorescence. (**B**) The spectral sensitivity of the iCCD drops off significantly beyond 800 nm (note the Log y-axis) as the image intensifier of the iCCD employs a Gen III VIH ($360 - 910$ nm) photocathode which (together with the grating) determines the spectral sensitivity of the detector. The BChl *a* Q_y emission is affected by the spectral sensitivity of the gated iCCD spectrometer. The emission spectra in (A) were corrected for the spectral sensitivity of the iCCD, but as the sensitivity in this region drops off significantly, the spectral shape should be treated with caution.

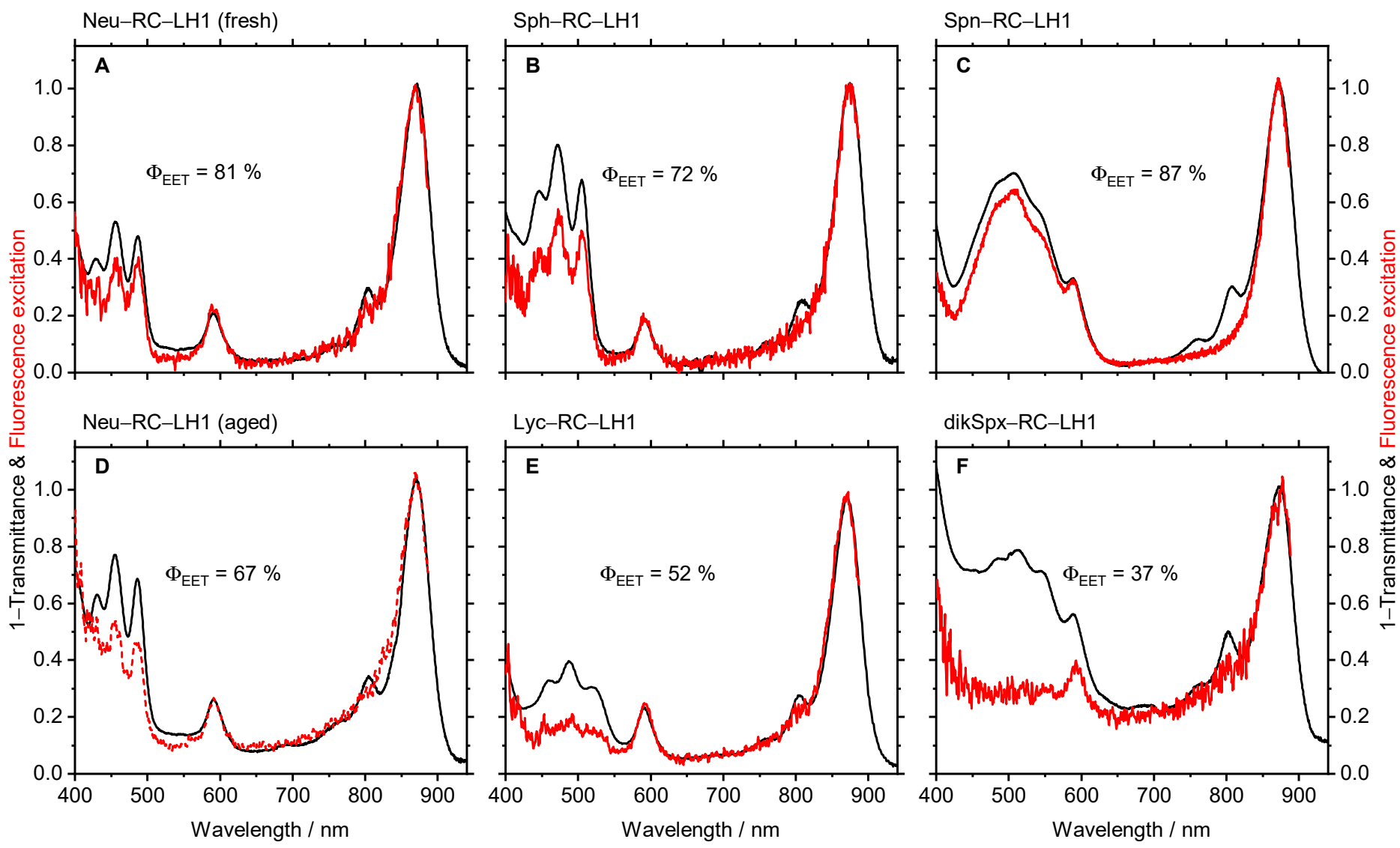

Fig. S18: 1–transmittance (black line) and fluorescence excitation (red line) spectra of RC–LH1 complexes containing Neu (**A**), Sph (**B**), Spn (**C**), Lyc (**E**), and dikSpx (**F**). The overall exciton energy transfer efficiency (Φ_{EET}) is displayed in each panel. We note that these spectra were measured within hours of purification and found that Φ_{EET} values obtained on older Neu–RC–LH1 samples had dropped to 67 % (**D**).

Table S1: Crt-to-BChl Exciton Energy Transfer (EET) efficiencies and associated parameters. S_1 lifetimes in LH1 complex, $\tau_{S_1}^{LH1}$ (see Fig. 2) and solvent ($\tau_{S_1}^{Sol}$) from Refs. [23–25] are used to estimate EET efficiency from $S_1 \rightarrow Q_y$ using $\Phi_{S_1 \rightarrow Q_y} = \left(1 - \tau_{S_1}^{LH1}/\tau_{S_1}^{Sol}\right) \times 100$, as described previously [23, 24]. The triplet yield Φ_T was calculated using data from the transient absorption spectra in Fig. 2 and published extinction coefficients [11]. The overall Crt-to-BChl *a* EET efficiency, Φ_{EET}, was determined by comparing 1-transmittance and fluorescence excitation spectra in Fig. S18, as described previously [22, 26–29]. The $S_2 \rightarrow Q_x$ EET efficiency, Φ_{Q_x} are best-guess estimates obtained by scaling spectral overlap factors reported in Ref. [23] with the experimental value for rhodopin glucoside ($N = 11$) [30]. These values are used to estimate the efficiency of energy transfer *via* the triplet pairs, $\eta\Phi_T$. Errors are given in parentheses, estimated from fitting errors, 2σ spread or from variability in the literature.

	S$_1$ lifetime (ps)			Triplet yield (%)		EET efficiency (%)		
						$S_2 \rightarrow Q_x \rightarrow Q_y$	$S_1 \rightarrow Q_y$	$S_2 \rightarrow (T \cdot\cdot T) \rightarrow Q_y$
	$\tau_{S_1}^{LH1}$	$\tau_{S_1}^{sol}$	Ref.	Φ_T	Φ_{EET}	Φ_{Q_x}	$\Phi_{S_1 \rightarrow Q_y}$	$\eta\Phi_T$
Neu	4.6 (0.4)	24	[23]	27 (5)	81 (4.6)	21 (10)	81 (2)	18 (8)
Sph	5.3 (0.6)	9.3	[24]	8 (4)	72 (2.8)	44 (10)	43 (6)	7 (4)
Spn	2.2 (0.4)	6	[23]	14 (4)	87 (3)	64 (10)	63 (7)	9 (6)
Lyc	4.0 (0.5)	4.7	[24]	3 (5)	52 (5)	51 (10)	15 (10)	
dikSpx	1.7 (0.4)	1.5	[25]		37 (5)			

Table S2: Extinction coefficients (ϵ) of Crt T_1 and S_2 and BChl *a* T_1, transient signal intensities of excited state absorption (ESA) of Crt T_1 ($\Delta t = 100\,ps$) and BChl *a* T_1 ($\Delta t = 2\,ns$) and initial ground state bleach (GSB) of Crt S_2 for RC–LH1 complexes after Crt S_2 excitation by TA spectroscopy, estimated triplet quantum yields (Φ_T) of Crt and BChl *a* generated *via* singlet fission.

RC–LH1 complex	ϵ^a Crt T_1 ($M^{-1}\,cm^{-1}$)	ϵ^b Crt S_2 ($M^{-1}\,cm^{-1}$)	ϵ^c BChl *a* T_1 ($M^{-1}cm^{-1}$)	ESA‡ Crt T_1 (ΔOD)	GSB§ Crt S_2 (ΔOD)	ESA‡ BChl *a* T_1 (ΔOD)	Φ Crt T_1 (%)	Φ BChl *a* T_1 (%)
Neu	2.74E5	1.594E5	1.036E4	2.36E-3	-5.10E-3	8.60E-5	27.0	26.0
Sph	3.09E5	1.736E5	1.036E4	5.68E-4	-3.80E-3	1.60E-5	8.4	7.1
Spn	6.06E4	1.220E5	1.036E4	2.37E-4	-3.50E-3	8.00E-5	13.6	26.9
Lyc	4.95E5	1.815E5	1.036E4	7.67E-5	-8.12E-4	1.00E-6	3.5	2.2

[a] from Ref. [11], [b] from Ref. [31], [c] from Ref. [10], ‡ ESA is for excited state absorption, § GSB is for ground state bleach.

Table S3: Crt S_1 energies estimated using different reported methods. Crt S_1 energies have been reported using different experimental methods. The generally accepted Spn S_1 energy is 1.61 eV extracted from transient absorption spectroscopy [23, 32], but this energy seems inconsistent with our findings in the main text (Fig. 4B). We, therefore, re-examine the estimated Spn S_1 energy. The generally accepted Neu and Sph S_1 energies were determined by using fluorescence spectroscopy [33]. When we use the reported linear regression of S_1 energies with respect to the conjugation lengths ($E(S_1) = 220946 \times \frac{1}{2N+1} + 3681$) we estimate the Spn S_1 energy to be 1.70 eV ($N = 10.5$). The S_1 energies of Crt molecules with longer conjugation lengths were also predicted by using S_1 lifetimes and the energy gap-law [34, 35]. Using this energy gap-law we estimate the Spn S_1 energy to be 1.70 eV ($\tau = 6$ ps [23]). Crt S_1 energies were also estimated using transient absorption spectroscopy [23], resulting in a Spn S_1 energy of 1.63 eV. Zigmantas *et al.* used the same technique and reported the energy to be about 1.61 eV [32]. Comparison of the two methods shows that transient absorption (TA)-calculated energies show a blue shift compared with the fluorescence- or energetic gap-law-calculated values. Indeed, the TA-obtained value matches the fluorescence 0–1 peak instead of the 0–0 peak. This inconsistency is interesting but we leave further discussion of it to later work and note only that the Spn S_1 energy follows the conjugation length trend of other Crts, despite the presence of an ICT state.

method	TA spectroscopy [23, 32]	fluorescence spectroscopy [33]<br>energy gap law with S_1 lifetimes [34, 35]
Crt	S_1 energy (cm^{-1})	
Neu	14170	15300
Sph	13160	14202
Spn	12800	13724
Lyc	–	13394
rhodopin glucoside	12400	–
Spx	–	11926

References

[1] Kakitani, Y. *et al.* Conjugation-length dependence of the t1 lifetimes of carotenoids free in solution and incorporated into the lh2, lh1, rc, and rc-lh1 complexes: possible mechanisms of triplet-energy dissipation. *Biochemistry* **46**, 2181–2197 (2007).

[2] Richert, S., Tait, C. E. & Timmel, C. R. Delocalisation of photoexcited triplet states probed by transient epr and hyperfine spectroscopy. *Journal of Magnetic Resonance* **280**, 103–116 (2017).

[3] Bayliss, S. L. *et al.* Spin signatures of exchange-coupled triplet pairs formed by singlet fission. *Physical Review B* **94**, 045204 (2016).

[4] Angerhofer, A., Bornhäuser, F., Gall, A. & Cogdell, R. Optical and optically detected magnetic resonance investigation on purple photosynthetic bacterial antenna complexes. *Chemical physics* **194**, 259–274 (1995).

[5] Scholes, G. D. Correlated pair states formed by singlet fission and exciton-exciton annihilation. *Journal of Physical Chemistry A* **119**, 12699–12705 (2015).

[6] Merrifield, R. E. Magnetic effects on triplet exciton interactions. *Pure and Applied Chemistry* **27**, 481–498 (1971).

[7] Johnson, R. C. & Merrifield, R. E. Effects of magnetic fields on the mutual annihilation of triplet excitons in anthracene crystals. *Physical Review B* **1**, 896–902 (1970).

[8] Bayliss, S. L. *et al.* Site-selective measurement of coupled spin pairs in an organic semiconductor. *Proceedings of the National Academy of Sciences* **115**, 5077–5082 (2018).

[9] Rondonuwu, F. S., Watanabe, Y., Fujii, R. & Koyama, Y. A first detection of singlet to triplet conversion from the $1^1\mathrm{B}_\mathrm{u}^-$ to the $1^3\mathrm{A}_\mathrm{g}$ state and triplet internal conversion from the $1^3\mathrm{A}_\mathrm{g}$ to the $1^3\mathrm{B}_\mathrm{u}$ state in carotenoids: Dependence on the conjugation length. *Chemical Physics Letters* **376**, 292–301 (2003).

[10] Borland, C. F., McGarvey, D. J., Truscott, T. G., Codgell, R. J. & Land, E. J. Photophysical studies of bacteriochlorophyll a and bacteriopheophytin a - singlet oxygen generation. *Journal of Photochemistry and Photobiology, B: Biology* **1**, 93–101 (1987).

[11] Cogdell, R. J., Land, E. J. & Truscott, T. G. The triplet extinction coefficients of some bacterial carotenoids. *Photochemistry and Photobiology* **38**, 723–725 (1983).

[12] Kosumi, D., Horibe, T., Sugisaki, M., Cogdell, R. J. & Hashimoto, H. Photoprotection mechanism of light-harvesting antenna complex from purple bacteria.

Journal of Physical Chemistry B **120**, 951–956 (2016).

[13] Jaumot, J., Gargallo, R., de Juan, A. & Tauler, R. A graphical user-friendly interface for MCR-ALS: a new tool for multivariate curve resolution in MATLAB. *Chemometrics and Intelligent Laboratory Systems* **76**, 101–110 (2005).

[14] Jaumot, J., de Juan, A. & Tauler, R. MCR-ALS GUI 2.0: New features and applications. *Chemometrics and Intelligent Laboratory Systems* **140**, 1–12 (2015).

[15] Monger, T. G., Cogdell, R. J. & Parson, W. W. Triplet states of bacteriochlorophyll and carotenoids in chromatophores of photosynthetic bacteria. *Biochimica et Biophysica Acta (BBA) - Bioenergetics* **449**, 136–153 (1976).

[16] Niedzwiedzki, D. M. & Blankenship, R. E. Singlet and triplet excited state properties of natural chlorophylls and bacteriochlorophylls. *Photosynthesis Research* **106**, 227–238 (2010).

[17] Hartwich, G., Scheer, H., Aust, V. & Angerhofer, A. Absorption and ADMR studies on bacterial photosynthetic reaction centres with modified pigments. *Biochimica et Biophysica Acta (BBA)-Bioenergetics* **1230**, 97–113 (1995).

[18] Tobita, S., Kaizu, Y., Kobayashi, H. & Tanaka, I. Study of higher excited singlet states of zinc (II)-tetraphenylporphin. *The Journal of Chemical Physics* **81**, 2962–2969 (1984).

[19] Yu, J. *et al.* Carotenoid singlet fission reactions in bacterial light harvesting complexes as revealed by triplet excitation profiles. *Journal of the American Chemical Society* **139**, 15984–15993 (2017).

[20] Uragami, C. *et al.* Photoprotective mechanisms in the core lh1 antenna pigment-protein complex from the purple photosynthetic bacterium, rhodospirillum rubrum. *Journal of Photochemistry and Photobiology A: Chemistry* **400**, 112628 (2020).

[21] Snellenburg, J. J., Laptenok, S., Seger, R., Mullen, K. M. & van Stokkum, I. H. M. Glotaran: A Java-based graphical user interface for the R package TIMP. *Journal of Statistical Software* **49**, 1–22 (2012).

[22] Niedzwiedzki, D. M., Swainsbury, D. J. K., Martin, E. C., Hunter, C. N. & Blankenship, R. E. Origin of the S* excited state feature of carotenoids in light-harvesting complex 1 from purple photosynthetic bacteria. *Journal of Physical Chemistry B* **121**, 7571–7585 (2017).

[23] Cong, H. *et al.* Ultrafast time-resolved carotenoid to-bacteriochlorophyll energy transfer in LH2 complexes from photosynthetic bacteria. *The Journal of Physical Chemistry B* **112**, 10689–10703 (2008).

[24] Zhang, J.-P. *et al.* Mechanism of the carotenoid-to-bacteriochlorophyll energy transfer via the S_1 state in the LH2 complexes from purple bacteria. *The Journal of Physical Chemistry B* **104**, 3683–3691 (2000).

[25] Šlouf, V. *et al.* Carotenoid to bacteriochlorophyll energy transfer in the RC-LH1-PufX complex from Rhodobacter sphaeroides containing the extended conjugation keto-carotenoid diketospirilloxanthin. *Photosynthesis research* **135**, 33–43 (2018).

[26] Akahane, J., Rondonuwu, F. S., Fiedor, L., Watanabe, Y. & Koyama, Y. Dependence of singlet-energy transfer on the conjugation length of carotenoids reconstituted into the LH1 complex from Rhodospirillum rubrum G9. *Chemical Physics Letters* **393**, 184–191 (2004).

[27] Chi, S. C. *et al.* Assembly of functional photosystem complexes in Rhodobacter sphaeroides incorporating carotenoids from the spirilloxanthin pathway. *Biochimica et Biophysica Acta - Bioenergetics* **1847**, 189–201 (2015).

[28] Noguchi, T., Hayashi, H. & Tasumi, M. Factors controlling the efficiency of energy transfer from carotenoids to bacteriochlorophyll in purple photosynthetic bacteria. *BBA - Bioenergetics* **1017**, 280–290 (1990).

[29] Sutherland, G. A., Qian, P., Hunter, C. N., Swainsbury, D. J. & Hitchcock, A. Engineering purple bacterial carotenoid biosynthesis to study the roles of carotenoids in light-harvesting complexes. *Methods in Enzymology* **674**, 137–184 (2022).

[30] Macpherson, A. N., Arellano, J. B., Fraser, N. J., Cogdell, R. J. & Gillbro, T. Efficient energy transfer from the carotenoid S_2 state in a photosynthetic light-harvesting complex. *Biophysical Journal* **80**, 923–930 (2001).

[31] Sashima, T., Koyama, Y., Yamada, T. & Hashimoto, H. The $1B_u^+$, $1B_u^-$, and $2A_g$-energies of crystalline lycopene, β-carotene, and mini-9-β-carotene as determined by resonance-Raman excitation profiles: dependence of the B_u-state energy on the conjugation length. *The Journal of Physical Chemistry B* **104**, 5011–5019 (2000).

[32] Zigmantas, D. *et al.* Effect of a conjugated carbonyl group on the photophysical properties of carotenoids. *Physical Chemistry Chemical Physics* **6**, 3009–3016 (2004).

[33] Fujii, R., Onaka, K., Kuki, M., Koyama, Y. & Watanabe, Y. The $2A_g^-$ energies of all-trans-neurosporene and spheroidene as determined by fluorescence spectroscopy. *Chemical physics letters* **288**, 847–853 (1998).

[34] Frank, H. A. *et al.* Spectroscopic properties of spheroidene analogs having different extents of π-electron conjugation. *The Journal of Physical Chemistry A* **101**, 149–157 (1997).

[35] Chynwat, V. & Frank, H. A. The application of the energy gap law to the S_1 energies and dynamics of carotenoids. *Chemical physics* **194**, 237–244 (1995).
